# Aldehyde dehydrogenase 2 preserves kidney function by countering acrolein-induced metabolic and mitochondrial dysfunction

**DOI:** 10.1172/jci.insight.179871

**Published:** 2024-10-08

**Authors:** Szu-Yuan Li, Ming-Tsun Tsai, Yu-Ming Kuo, Hui-Min Yang, Zhen-Jie Tong, Hsiao-Wei Cheng, Chih-Ching Lin, Hsiang-Tsui Wang

**Affiliations:** 1 Division of Nephrology, Department of Medicine, Taipei Veterans General Hospital, Taipei, Taiwan.; 2 School of Medicine and; 3 Institute of Pharmacology, College of Medicine, National Yang Ming Chiao Tung University, Taipei, Taiwan.; 4 Institute of Food Safety and Health Risk Assessment, National Yang Ming Chiao Tung University, Taipei, Taiwan.; 5 Doctor Degree Program in Toxicology, Kaohsiung Medical University, Kaohsiung, Taiwan.

**Keywords:** Metabolism, Nephrology, Chronic kidney disease, Fibrosis, Mitochondria

## Abstract

The prevalence of chronic kidney disease (CKD) varies by race because of genetic and environmental factors. The Glu504Lys polymorphism in aldehyde dehydrogenase 2 (ALDH2), commonly observed among East Asian people, alters the enzyme’s function in detoxifying alcohol-derived aldehydes, affecting kidney function. This study investigated the association between variations in ALDH2 levels within the kidney and the progression of kidney fibrosis. Our clinical data indicate that diminished ALDH2 levels are linked to worse CKD outcomes, with correlations between ALDH2 expression, estimated glomerular filtration rate, urinary levels of acrolein — an aldehyde metabolized by ALDH2 — and fibrosis severity. In mouse models of unilateral ureteral obstruction and folic acid nephropathy, reduced ALDH2 levels and elevated acrolein were observed in kidneys, especially in ALDH2 Glu504Lys–knockin mice. Mechanistically, acrolein modifies pyruvate kinase M2, leading to its nuclear translocation and coactivation of HIF-1α, shifting cellular metabolism to glycolysis, disrupting mitochondrial function, and contributing to tubular damage and the progression of kidney fibrosis. Enhancing ALDH2 expression through adeno-associated virus vectors reduced acrolein and mitigated fibrosis in both WT and Glu504Lys-knockin mice. These findings underscore the potential therapeutic significance of targeting the dynamic interaction between ALDH2 and acrolein in CKD.

## Abstract 2

Targeting the interaction between aldehyde dehydrogenase 2 and acrolein, a byproduct of lipid peroxidation, may offer a therapeutic strategy for chronic kidney disease (CKD).

## Graphical abstract

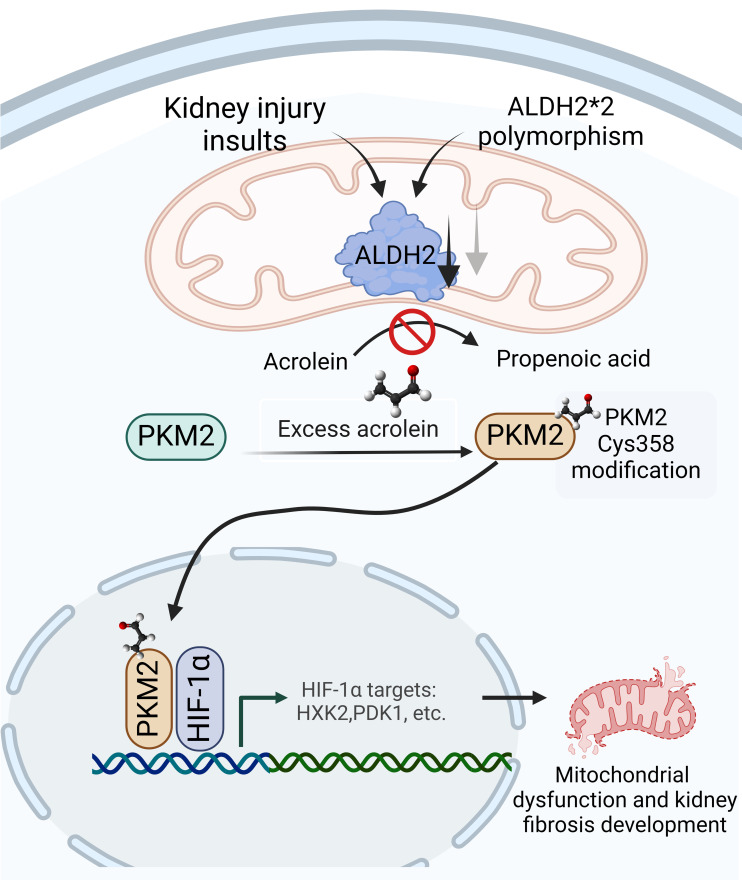

## Introduction

Chronic kidney disease (CKD) is characterized by progressing kidney fibrosis and a gradual decline in kidney function. Treating CKD involves addressing the primary cause of kidney injuries and managing standard processes contributing to ongoing nephron loss. Alterations in lipid metabolism are crucial in initiating and propagating kidney disease, especially in diabetic kidney disease (DKD) (1, 2), where disruptions in tubular lipid metabolism result from imbalances in fatty acid uptake, metabolism, and synthesis. Impaired tubular lipid metabolism subsequently leads to mitochondrial dysfunction, cellular apoptosis, and the development and progression of kidney fibrosis (3, 4).

Lipotoxicity may arise because of cytotoxic aldehydes generated during lipid peroxidation triggered by oxidative stress (5). Hydroxyl radical attacks on fatty acyl chains lead to lipid peroxidation, producing α,β-unsaturated lipid aldehydes, such as 4-hydroxynonenal, 4-hydroxyhexenal, malondialdehyde, and acrolein (6, 7). These harmful aldehydes interact with intracellular macromolecules, including proteins and DNA, potentially leading to gene mutations and cellular damage (8, 9). Acrolein stands out among aldehydes as exceptionally toxic to cells owing to its high electrophilicity, which promotes rapid reactions with nucleophilic molecules. This reactivity facilitates the formation of adducts, disrupting cellular processes. Additionally, acrolein directly inhibits detoxifying enzymes, such as aldehyde dehydrogenase 2 (ALDH2), creating a vicious cycle that exacerbates its levels in diseased states (10, 11). Previous small-scale studies have shown that patients with CKD often have elevated serum acrolein levels (12, 13). Our investigation also revealed that administering acrolein scavengers effectively improves metabolic disturbances, kidney function, and acrolein-protein conjugate (Acr-PC) production in a mouse DKD model (14).

ALDH2, a member of the ALDH superfamily, is present in the mitochondrial matrix and highly expressed in vital organs like the heart, liver, and kidney (15). As an allosteric tetramer, this enzyme is crucial in oxidizing endogenous and exogenous aldehydes into their respective acids (16). Several studies investigated the role of ALDH2 in various kidney injuries, showing its protective function in acute kidney injury (AKI) through autophagy-related gene activation (17–19). Tang et al. conducted research to analyze transcriptome data from animal models of kidney disease. Their findings suggest that lower levels of ALDH2 are linked to the development of kidney fibrosis (20). Furthermore, the prevalent ALDH2 Glu504Lys polymorphism (ALDH2*2) in East Asian people, which exhibits reduced catalytic activity, was identified as an independent risk factor for decreased kidney function in Japanese patients with hypertension (21). These findings suggest that ALDH2 may be involved in detoxifying and clearing harmful aldehydes in the kidney, though the underlying molecular mechanisms remain to be elucidated.

This study aims to elucidate the influence of kidney ALDH2 expression on acrolein levels and the development of kidney fibrosis. Additionally, we investigate the potential of enhancing kidney ALDH2 expression to mitigate kidney damage.

## Results

### Correlation of ALDH2 expression with kidney fibrosis, urinary acrolein levels, and kidney outcomes.

**Figure 1:**
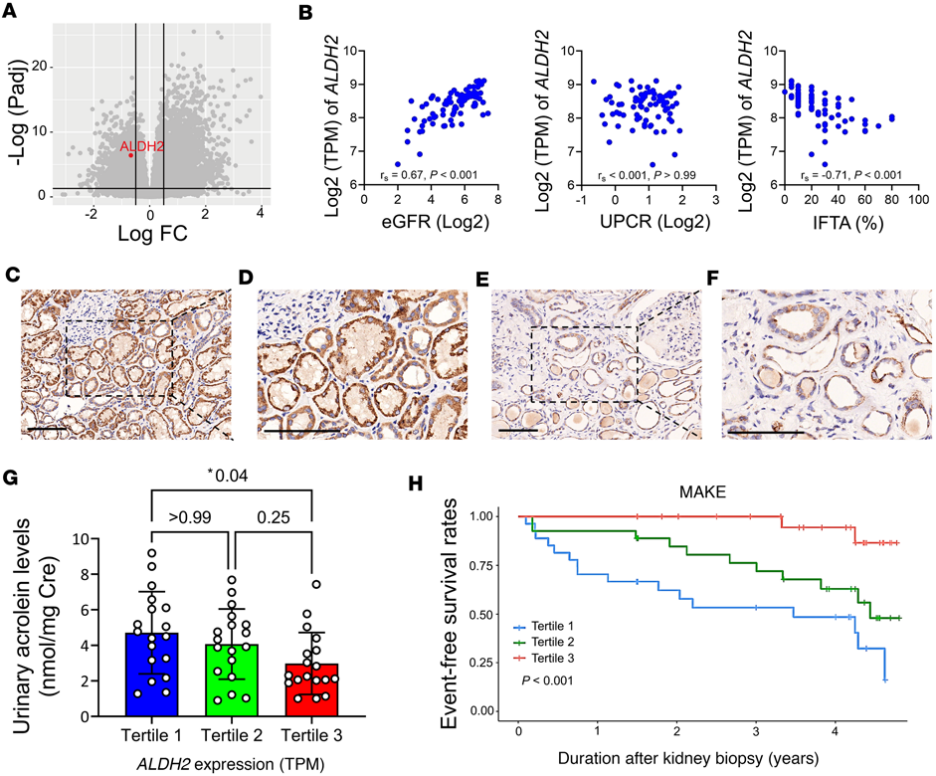
The diminished expression of ALDH2 in kidney tissues is correlated with adverse kidney outcomes in individuals with CKD. (**A**) The volcano plot illustrates gene expression changes, indicating *ALDH2* downregulation in fibrotic kidney disease compared with nonfibrotic cases. The *x* axis represents log_2_ fold-change, and the *y* axis represents –log_10_ (*P* values). (**B**) Scatterplots depict the correlation between kidney *ALDH2* mRNA levels and estimated glomerular filtration rate (eGFR, the left panel), proteinuria (the middle panel), and the extent of interstitial fibrosis and tubular atrophy (IFTA, the right panel). Correlation coefficients (r_s_) and *P* values are displayed. (**C**–**F**) Immunohistochemical staining of kidney biopsies from CKD versus healthy controls showed strong ALDH2 positivity in normal tubular epithelia (**C**), contrasting with reduced expression in advanced CKD (**E**). Enlarged views are displayed on the right (**D** and **F**). (Original magnifications of ×200 in **C** and **E** and ×400 in **D** and **F**.) Scale bar: 100 μm. (**G**) Urinary acrolein levels in CKD were assessed based on kidney *ALDH2* mRNA tertiles. Acrolein levels increased with declining *ALDH2* expression, with tertiles at 238.3 (197.7–273.2), 352.8 (333.3–374.6), and 427.8 (408.5–486.2) transcripts per million (TPM). (**H**) Major adverse kidney events (MAKEs), stratified by kidney *ALDH2* tertiles, showed a significant association in Kaplan-Meier survival curves, determined by the log-rank test. An MAKE was defined as a greater than 40% decline in eGFR, kidney failure, or death. *ALDH2*, aldehyde dehydrogenase 2; CKD, chronic kidney disease; UPCR, urine protein creatinine ratio.

To assess the expression of ALDH2 in the kidney, we analyzed RNA-Seq data from microdissected tubulointerstitial sections of 81 human kidney samples from the Taipei Renal Transcriptomics and Outcomes Investigation cohort (22). Detailed demographic and clinical traits of the participants are provided in Supplemental Tables 1 and 2; supplemental material available online with this article; https://doi.org/10.1172/jci.insight.179871DS1 Using a greater than 10% interstitial fibrosis and tubular atrophy (IFTA) threshold to define kidney fibrosis, we observed a significant decrease in ALDH2 expression in fibrotic kidneys (Figure 1A). Further analysis revealed a direct correlation between kidney ALDH2 mRNA levels and estimated glomerular filtration rate (eGFR) and an inverse association with the degree of IFTA (Figure 1B). Immunohistochemistry (IHC) staining on kidney biopsy specimens validated ALDH2 expression and location. In contrast with the robust and widespread ALDH2 staining observed in tubular epithelia of individuals with preserved kidney function and low fibrosis (Figure 1, C and D), reduced staining intensity of ALDH2 was noted in kidney samples from fibrotic kidney tissues (Figure 1, E and F).

To explore the influence of kidney ALDH2 expression on aldehyde metabolism, we analyzed urinary acrolein concentration; our results revealed that individuals in the lowest tertile of kidney ALDH2 expression exhibited the highest urinary acrolein concentration (Figure 1G). During a median 3.5-year follow-up, major adverse kidney events (MAKEs) occurred in 29 participants. Stratifying patients into tertiles based on kidney *ALDH2* mRNA levels revealed significant differences in outcomes among these groups (*P* < 0.001, Figure 1H). Diminished kidney *ALDH2* mRNA correlated with an elevated risk of experiencing MAKEs in Cox proportional regression analysis. This association remained significant after adjusting for parameters, including histopathological diagnosis, but lost significance when considering baseline eGFR (Supplemental Table 3). These findings indicate that reduced kidney ALDH2 is linked to chronic histological injuries, accumulation of acrolein, and an unfavorable prognosis in patients with CKD.

### ALDH2 reduction and acrolein-protein conjugate increase in animal kidney fibrotic models.

**Figure 2:**
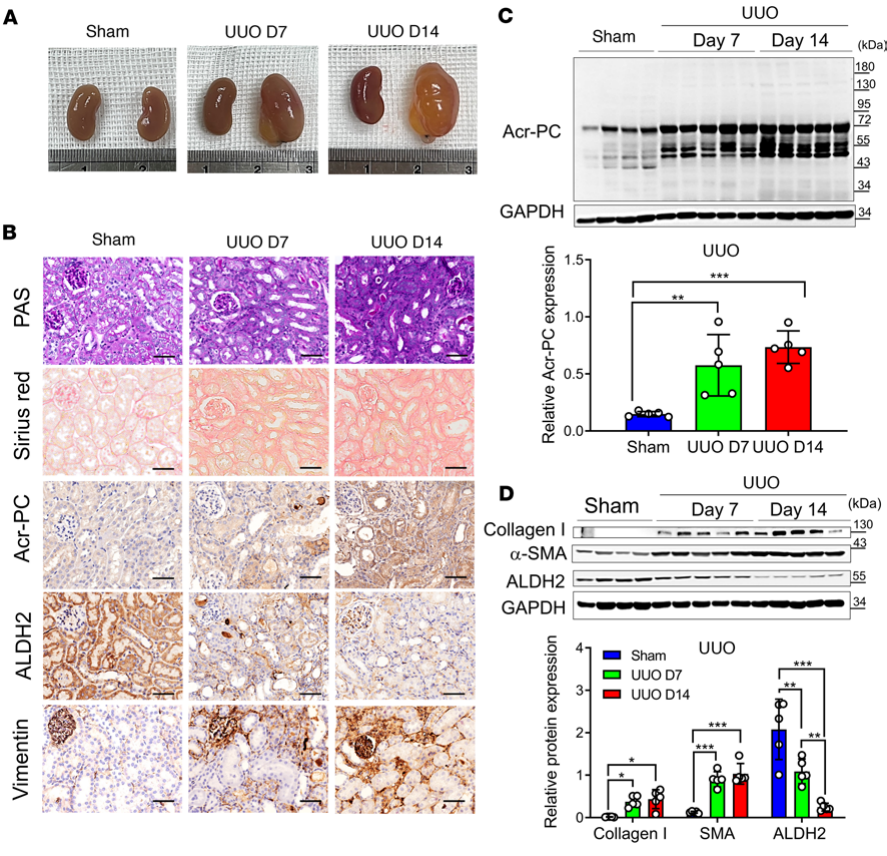
Analysis of Acr-PCs, ALDH2 expression, and fibrosis markers in Aldh2*1 mice after UUO. UUO surgery was conducted in WT mice (*n* = 5), and the obstructed kidneys were collected at 7 and 14 days after surgery. (**A**) Representative kidney gross appearance from WT mice at day 7 or 14 after UUO. (**B**) The first panel displays periodic acid–Schiff (PAS) staining to assess morphological alterations in kidney tissues. The second panel exhibits Sirius red staining to evaluate the extent of kidney fibrosis on day 7 and 14 after UUO. The third through fifth panels present immunohistochemistry depicting Acr-PC levels, ALDH2, and Vimentin in kidney tissues. Scale bar: 50 μm. (**C**) The upper panel illustrates Western blot analysis of Acr-PC in kidney tissues of mice at day 7 and 14 after UUO, with quantification of these proteins shown in the lower panel. (**D**) The upper panel presents Western blot analysis of collagen 1, α–smooth muscle actin (α-SMA), and ALDH2 in kidney tissues of mice at day 7 and 14 after UUO, with quantification of these proteins shown in the lower panel. Data are represented as mean ± SD. Statistical significance was determined using Kruskal-Wallis tests, with 2-tailed *P* values displayed. *P < 0.05, **P < 0.01, ***P < 0.001 compared with the vehicle control group. Acr-PCs, acrolein-protein conjugates; ALDH2, aldehyde dehydrogenase 2; WT, wild-type; UUO, unilateral ureteral obstruction.

**Figure 3:**
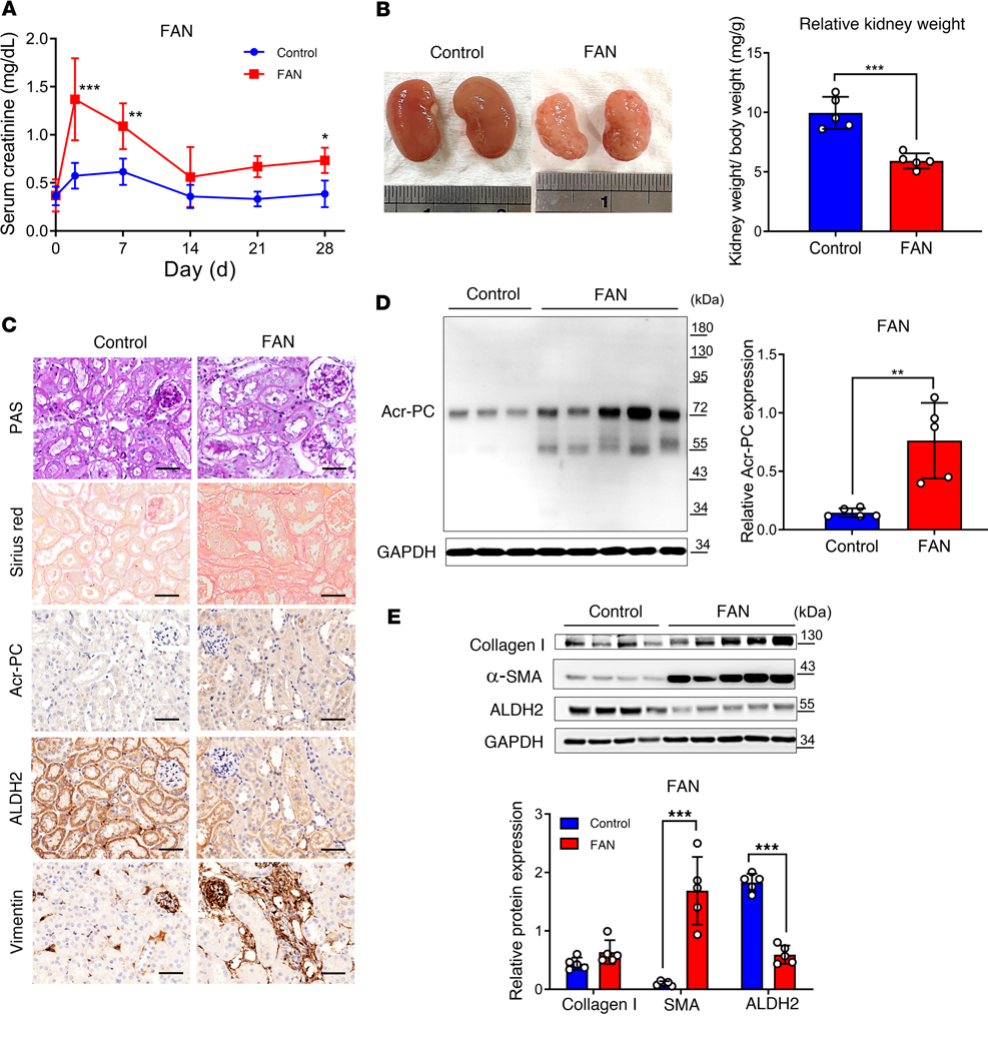
Assessment of Acr-PCs, ALDH2 expression, and fibrosis markers in Aldh2*1 mice after FAN. WT mice (*n* = 5) received intraperitoneal injections of folic acid (FA) (225 mg/kg in 300 mM NaHCO_3_) and were subsequently sacrificed on day 28. (**A**) Measurement of serum creatinine (sCr) of mice on day 0, 3, 7, 14, 21, and 28 after FA injection. (**B**) Representative kidney gross appearance is displayed in the left panel, and the right panel presents a statistical analysis of the kidney-to-body weight ratio. (**C**) Periodic acid–Schiff (PAS) staining is shown in the first panel to assess morphological changes in kidney tissues. Sirius red staining to evaluate the kidney fibrosis area on day 28 after FA injection is depicted in the second panel. Immunohistochemistry for Acr-PCs, ALDH2, and Vimentin in kidney tissues is presented in the third through fifth panels. Scale bar: 50 μm. (**D**) The left panel shows Western blot analysis of Acr-PCs in kidney tissues of mice on day 28 after FA injection, with quantification of these proteins presented in the right panel. (**E**) The upper panel shows Western blot analysis of collagen 1, α–smooth muscle actin (α-SMA), and ALDH2 in kidney tissues of mice on day 28 after FA injection, with quantification of these proteins presented in the lower panel. Data are represented as mean ± SD. Statistical significance was determined using Mann-Whitney *U* tests, and 2-tailed *P* values are shown. *P < 0.05, **P < 0.01, ***P < 0.001 compared with the vehicle control group. Acr-PCs, acrolein-protein conjugates; ALDH2, aldehyde dehydrogenase 2; WT, wild-type; FAN, folic acid nephropathy.

To substantiate our clinical observation regarding the reduction of ALDH2 and acrolein accumulation in kidney disease progression, we utilized 2 kidney fibrosis models, the unilateral ureteral obstruction (UUO) model and folic acid nephropathy (FAN), in wild-type mice. In the UUO model, ureter ligation induced hydronephrosis, tubular injury, and interstitial fibrosis (Figure 2, A and B). Corresponding with our clinical observation using urinary acrolein as a surrogate marker, there was a significant rise in Acr-PCs observed in UUO kidney tubules (Figure 2, B and C). Western blot analysis of kidney homogenates revealed a substantial, time-dependent reduction in ALDH2 expression after UUO (Figure 2D). In the FAN model, administration of folic acid induced AKI progressing to CKD, with an elevation in serum creatinine on day 3, followed by partial recovery and the progression of interstitial fibrosis and kidney atrophy (Figure 3, A and B). Sirius red staining on day 28 demonstrated marked IFTA characterized by substantial collagen deposition (Figure 3C). Similar to the UUO model, folic acid–treated mice exhibited a substantial increase in Acr-PC levels and a reduction in ALDH2 expression (Figure 3, C–E).

### ALDH2-mutant mice exhibit increased vulnerability to kidney fibrosis.

To elucidate the causal relationship between diminished ALDH2 expression and the progression of kidney disease, we employed ALDH2 Glu504Lys–knockin mice in our animal experiments. The enzyme activity of both homozygous (Aldh2*2/*2) and heterozygous (Aldh2*1/*2) genotypes of the mutant ALDH2 was found to be 17%–38% of that in normal ALDH2 genotypes (23). We verified the reduction in protein expression and activity of ALDH2 in the liver and kidney of Aldh2*1/*2 or Aldh2*2/*2 mice (Supplemental Figure 1, A–D). Remarkably, mice with the Aldh2*2/*2 genotype displayed normal kidney development, exhibiting no significant differences in kidney histology or function (Supplemental Figure 1E).

**Figure 4:**
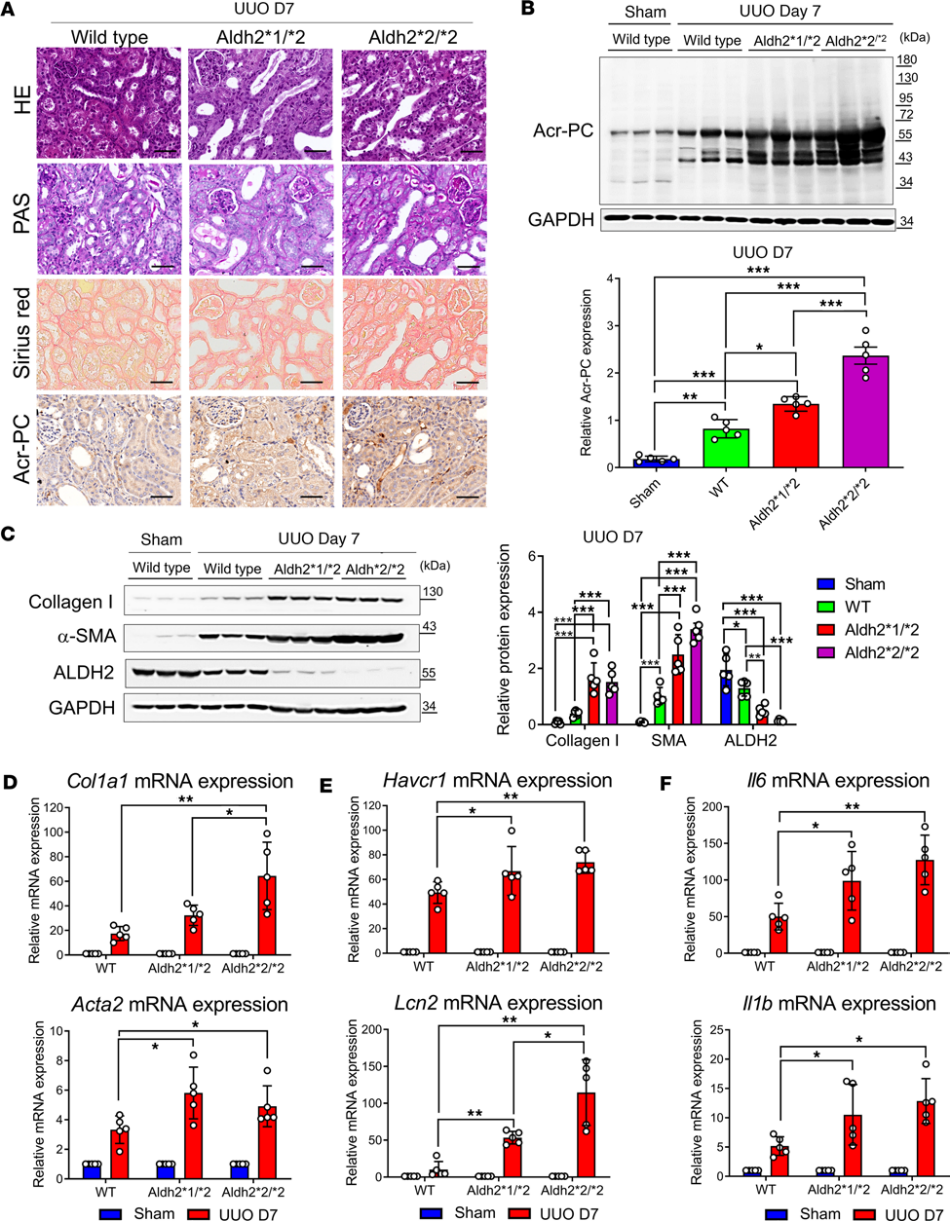
Evaluation of Acr-PCs, fibrosis markers, inflammatory cytokines, and kidney damage markers in Aldh2*1/*2 and Aldh2*2/*2 mice compared with Aldh2*1 mice after UUO. A UUO model was induced in WT, Aldh2*1/*2, and Aldh2*2/*2 mice (*n* = 5 for each group), and kidneys were collected 7 days after surgery. (**A**) H&E and periodic acid–Schiff (PAS) staining to assess morphological changes in kidney tissues (first and second panels). Sirius red staining evaluates the kidney fibrosis area on day 7 after UUO (third panel). Immunohistochemistry for Acr-PCs in kidney tissues is presented in the fourth panel. Scale bar: 50 μm. (**B**) The upper panel shows Western blot analysis of Acr-PC in kidney tissues, with quantification of these proteins shown in the lower panel. (**C**) The left panel shows Western blot analysis of collagen 1, α–smooth muscle actin (α-SMA), and ALDH2 in kidney tissues, with quantification of these proteins shown in the right panel. mRNA expression of (**D**) *Col1a1*, *Acta2*, (**E**) *Havcr1*, *Lcn2*, and (**F**) *Il6* and *Il1b* in kidney tissues was assessed using quantitative reverse transcription PCR analysis. Data are presented as mean ± SD. Statistical significance was determined using Kruskal-Wallis tests, and 2-tailed *P* values are shown. *P < 0.05, **P < 0.01, ***P < 0.001 compared with the WT group. Acr-PCs, acrolein-protein conjugates; ALDH2, aldehyde dehydrogenase 2; WT, wild-type; UUO, unilateral ureteral obstruction; *Acta2*, actin alpha 2; *Aldh2*, aldehyde dehydrogenase 2; *Col1a1*, collagen type I alpha 1 chain; *Havcr1*, hepatitis A virus cellular receptor 1 homolog; *Il6*, interleukin-6, *Il1b*, interleukin-1β; *Lcn2*, lipocalin-2.

The extent of kidney fibrosis in Aldh2 wild-type, Aldh2*1/*2, and Aldh2*2/*2 mice was assessed in the UUO model. Our results revealed higher interstitial fibrosis and Acr-PC levels in Aldh2*1/*2 and Aldh2*2/*2 mice compared with Aldh2 wild-type mice following UUO (Figure 4, A and B). Morphological observations of kidney fibrosis were supported by Western blot analysis and quantitative reverse transcription PCR (RT-PCR) of kidney tissues (Figure 4, C and D). Additionally, Aldh2*1/*2 and Aldh2*2/*2 mice exhibited elevated levels of kidney damage markers, such as neutrophil gelatinase-associated lipocalin and kidney injury molecule 1, as well as inflammatory cytokines IL-6 and IL-1β, compared with Aldh2 wild-type mice following UUO (Figure 4, E and F).

**Figure 5:**
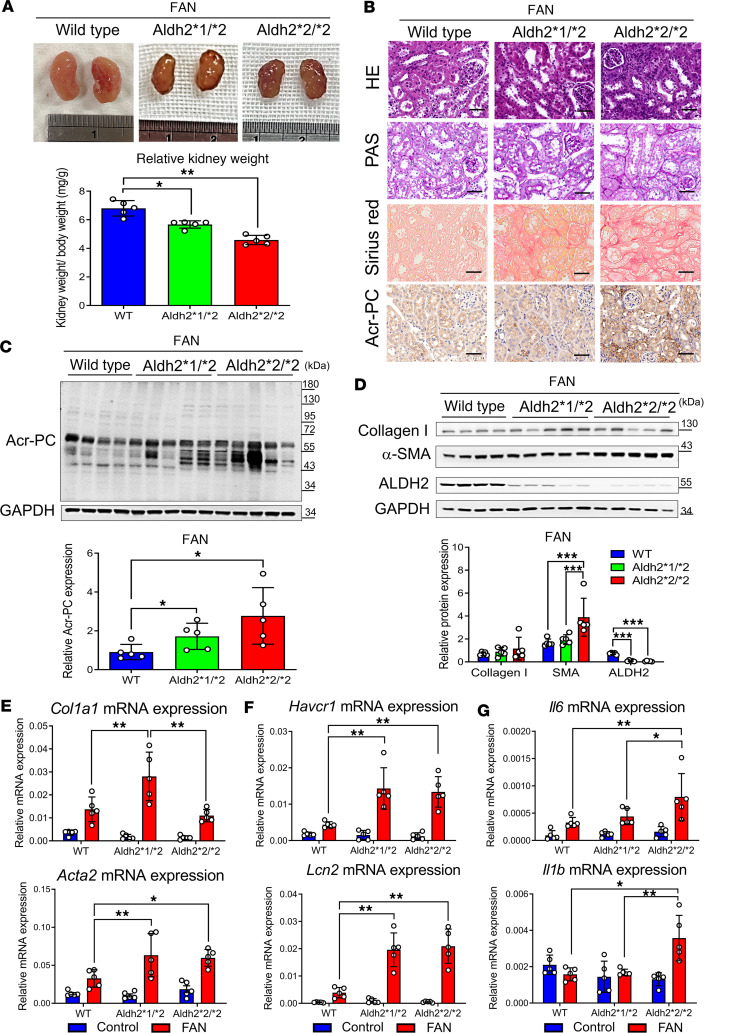
Assessment of Acr-PCs, fibrosis markers, inflammatory cytokines, and kidney damage markers in Aldh2*1/*2 and Aldh2*2/*2 mice compared with Aldh2*1 mice after FAN. WT, Aldh2*1/*2, and Aldh2*2/*2 mice (*n* = 5 for each group) were intraperitoneally administered folic acid (FA) (225 mg/kg in 300 mM NaHCO_3_) and sacrificed on day 28. (**A**) The upper panel displays the gross appearance of representative kidneys, while the lower panel presents the statistical analysis of the kidney-to-body weight ratio. (**B**) H&E and periodic acid–Schiff (PAS) staining are shown in the first and second panels to assess morphological changes in kidney tissues. Sirius red staining to evaluate the kidney fibrosis area on day 28 after FA injection is depicted in the third panel. Immunohistochemistry for Acr-PC in kidney tissues is presented in the fourth panel. Scale bar: 50 μm. The upper panel displays Western blot analysis of (**C**) Acr-PCs, (**D**) collagen 1, α–smooth muscle actin (α-SMA), and ALDH2 in kidney tissues of mice, with quantification of these proteins shown in the lower panel. mRNA expression of (**E**) *Col1a1*, *Acta2*, (**F**) *Havcr1*, *Lcn2*, and (**G**) *Il6* and *Il1b* in kidney tissues of mice was assessed using quantitative reverse transcription PCR analysis. Data are presented as mean ± SD. Statistical significance was determined using Kruskal-Wallis tests, and 2-tailed *P* values are shown. *P < 0.05, **P < 0.01, ***P < 0.001 compared with the WT group. Acr-PCs, acrolein-protein conjugates; ALDH2, aldehyde dehydrogenase 2; WT, wild-type; FAN, folic acid nephropathy; *Acta2*, actin alpha 2; *Aldh2*, aldehyde dehydrogenase 2; *Col1a1*, collagen type I alpha 1 chain; *Havcr1*, hepatitis A virus cellular receptor 1 homolog; *Il6*, interleukin-6, *Il1b*, interleukin-1β; *Lcn2*, lipocalin-2.

We also investigated the impact of folic acid–induced kidney injury in Aldh2*1/*2, Aldh2*2/*2, and Aldh2 wild-type mice. Our findings demonstrated more severe kidney atrophy in Aldh2*1/*2 and Aldh2*2/*2 mice 28 days after FAN (Figure 5A). sCr levels and body weight changes remained comparable among these groups in the 28 days following the injection of folic acid (Supplemental Figure 1, F and G). Similar to the UUO model, folic acid–induced interstitial fibrosis and upregulation of Acr-PC levels were more prominent in Aldh2*1/*2 and Aldh2*2/*2 mice than in Aldh2 wild-type mice (Figure 5, B–E, and Supplemental Figure 1, H and I). Similar to the UUO model, folic acid–induced kidney damage markers and upregulation of inflammatory cytokines were higher in Aldh2*1/*2 mice compared with their wild-type littermates (Figure 5, F and G).

### Acrolein induces pyruvate kinase M2 Cys358 modification and translocation from cytosol to cell nucleus and coactivates HIF-1α signaling.

**Figure 6:**
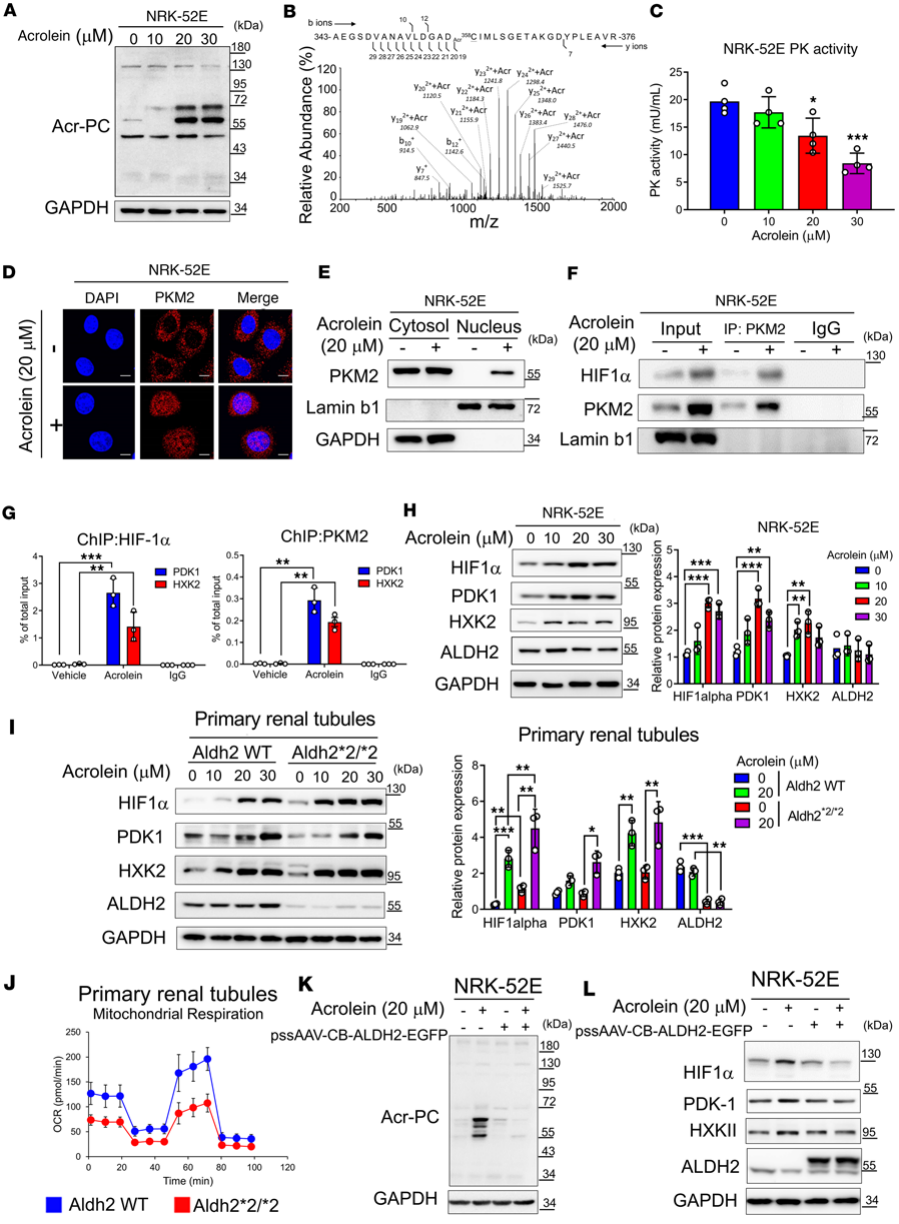
Acrolein-modified PKM2 shifts mitochondrial oxidative phosphorylation to aerobic glycolysis in NRK-52E cells and primary mouse renal tubular epithelial cells. (**A**–**C**) NRK-52E cells were exposed to acrolein (0–30 μM) for 24 hours. (**A**) Western blot analysis of Acr-PCs is presented. (**B**) Tandem mass spectrometry illustrating the acrolein-modified peptide in acrolein-treated NRK-52E cells. (**C**) Pyruvate kinase (PK) activity was determined. (**D**) Immunofluorescence staining of PKM2 in acrolein-treated NRK-52E cells. Scale bar: 10 μm. (**E**) Subcellular localization of PKM2 in acrolein-treated NRK-52E cells. Cells treated with acrolein (20 μM, 24 hours) were subjected to the Cell Fractionation Kit, followed by Western blot analysis. (**F**) Co-immunoprecipitation analysis of nuclear fractions prepared from acrolein-treated NRK-52E cells using an anti-PKM2 antibody or IgG antibody, followed by Western blot analysis. (**G**) Cells treated with acrolein (20 μM, 24 hours) were subjected to chromatin immunoprecipitation (ChIP) assays with antibodies against HIF-1α (the left panel), PKM2 (the right panel), or IgG, followed by real-time quantitative PCR for PDK1 and HXK2. (**H**) NRK-52E cells treated with acrolein (0–30 μM) for 24 hours were subjected to Western blot analysis with quantification. (**I**) Primary renal tubular epithelial cells isolated from Aldh2 WT or Aldh2*2/*2 mice treated with acrolein (0–30 μM) for 24 hours were subjected to Western blot analysis with quantification. (**J**) Oxygen consumption rate (OCR) was analyzed using the Seahorse XFe24 Metabolic Flux Analyzer. (**K** and **L**) ALDH2 overexpression in NRK-52E cells using AAV8-ALDH2-EGFP transient transfection for 24 hours followed by acrolein treatment (20 μM, 24 hours). Western blot analysis was performed. Data are presented as mean ± SD. Statistical significance was determined using Kruskal-Wallis tests, with 2-tailed *P* values indicated. *P < 0.05, **P < 0.01, ***P < 0.001 compared with vehicle treatment. Acr-PCs, acrolein-protein conjugates; PKM2, pyruvate kinase M2; HXK2, hexokinase 2; PDK1, pyruvate dehydrogenase kinase 1.

Given our clinical data and animal experiments suggesting that altered ALDH2 activity and subsequent acrolein accumulation exacerbate kidney disease, we employed a cell culture model to uncover the underlying mechanisms. Exposing NRK-52E cells to acrolein resulted in a dose-dependent increase in Acr-PC and pyruvate kinase M2 (PKM2) Cys358 modification (Figure 6, A and B, and Supplemental Table 4), reducing PKM2 activity (Figure 6C). Using confocal microscopy, we observed that acrolein-induced PKM2 Cys358 modification caused PKM2 to translocate from the cytosol to the nucleus, an observation verified by nuclear/cytosol fractionation (Figure 6, D and E). Since nuclear PKM2 has been reported as an HIF-1α cofactor in cancer cells (24), we examined this interaction in tubular cells. Our results verified a structural protein-protein interaction between PKM2 and HIF-1α in acrolein-treated NRK-52E cells (Figure 6F). Next, we tested whether acrolein-induced PKM2 nuclear translocation coactivates HIF-1α signaling in tubular cells. Through ChIP experiments, we verified that nuclear PKM2 binds to the promoter regions of HIF-1α downstream genes, including pyruvate dehydrogenase kinase 1 (PDK1) and hexokinase 2 (HXK2) (Figure 6G). We further verified that acrolein dose-dependently increased the expression of HIF-1α and its downstream genes (Figure 6H). We then used primary cultured mouse tubular cells from wild-type and Aldh2*2/*2 mice to examine the effect of acrolein on HIF-1α signaling. The results showed that, at a constant dose of acrolein, tubular cells from Aldh2*2/*2 mice were more sensitive to acrolein and exhibited more pronounced HIF-1α pathway signaling (Figure 6I). We also observed that primary renal tubular epithelial cells with the Aldh2*2/*2 genotype displayed diminished mitochondrial respiratory function compared with those with wild-type genotypes (Figure 6J). Finally, we used adeno-associated virus (AAV) vector to overexpress ALDH2 in tubular cells. Our data illustrated that overexpression of ALDH2 effectively reduced acrolein-induced Acr-PC production and HIF-1α activation (Figure 6, K and L). We also observed that acrolein triggered the production of intracellular and mitochondrial reactive oxygen species (ROS), lowered mitochondrial membrane potential, and reduced ATP production (Supplemental Figure 2, A–D). Using the Seahorse metabolic analyzer, we found that acrolein decreased maximal respiratory capacity and ATP turnover in cultured tubular NRK-52E cells (Supplemental Figure 2, E–G) and primary cultured mouse tubular cells (Supplemental Figure 2, H–J).

### ALDH2-mutant mice exhibit a more pronounced shift from mitochondrial respiration to glycolysis after kidney injury.

**Figure 7:**
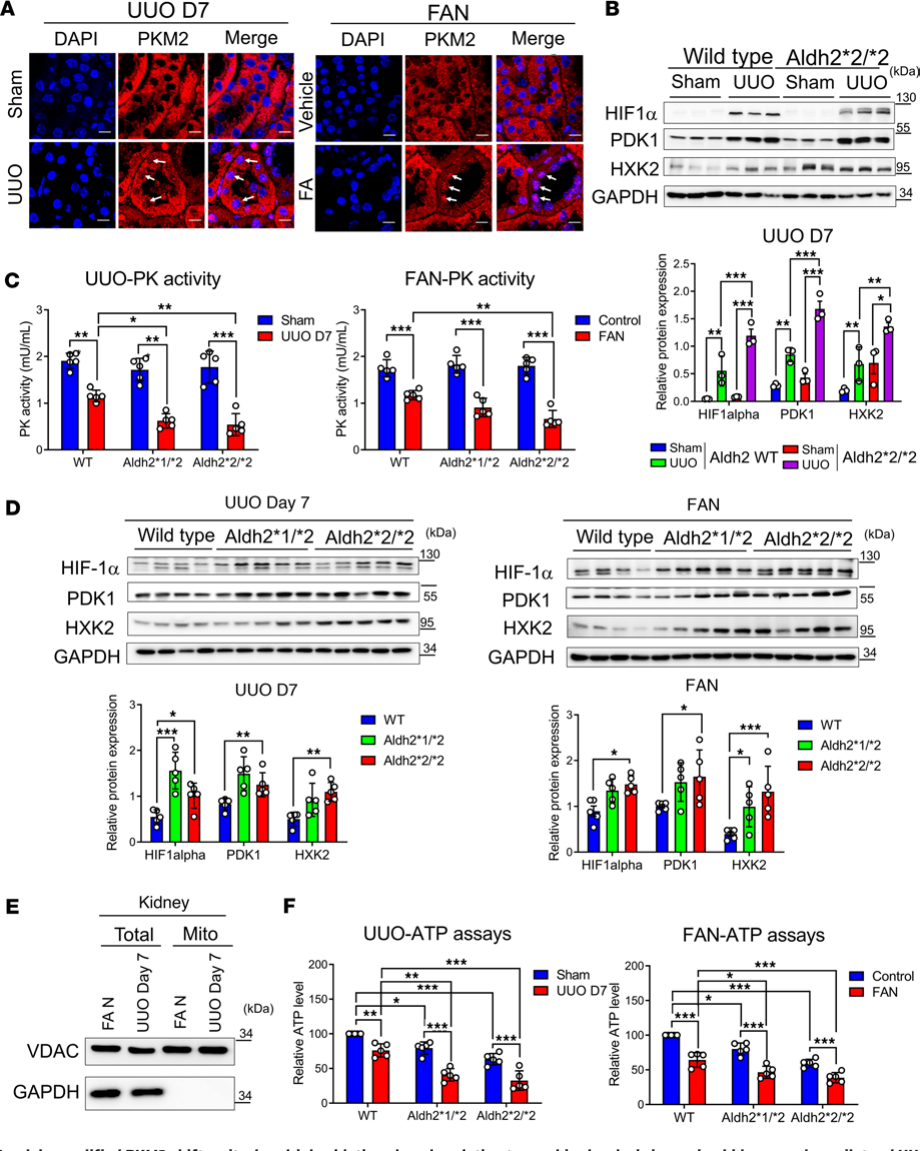
Acrolein-modified PKM2 shifts mitochondrial oxidative phosphorylation to aerobic glycolysis in murine kidneys under unilateral UUO or FAN. (**A**) Immunofluorescence staining of PKM2 in the kidney tissues of mice exposed to UUO on day 7 (the left panel) or folic acid (FA) injection on day 28 (the right panel). Nuclei were counterstained with DAPI. The white arrows indicate representative nuclear translocation of PKM2. Scale bar: 10 μm. (**B**) The upper panel displays Western blot analysis of HIF-1α, PDK-1, and HXK2 in kidney tissues of sham and UUO groups of Aldh2 wild-type (WT) and Aldh2*2/*2 mice, with quantification of these proteins shown in the lower panel. (**C**) Pyruvate kinase (PK) activity in the kidney tissues of mice exposed to UUO day 7 (the left panel) or FA injection day 28 (the right panel) was determined. (**D**) The upper panel displays Western blot analysis of HIF-1α, PDK-1, and HXK2 in kidney tissues of mice exposed to UUO on day 7 (left panel) or FA injection on day 28 (right panel), with quantification of these proteins shown in the lower panel. (**E**) Mouse kidney mitochondria were isolated. Western blot analysis of subcellular preparations (Total, total lysates; Mito, mitochondria) probed with antibodies specific for organelle-specific marker proteins: cytosol (GAPDH) and mitochondria (voltage-dependent anion channel, VDAC). (**F**) ATP content of mitochondria isolated from kidney tissues of mice exposed to UUO on day 7 (the left panel) or FA injection on day 28 (the right panel) was measured. Data are presented as mean ± SD. Statistical significance was determined using Kruskal-Wallis tests, with 2-tailed *P* values indicated. *P < 0.05, **P < 0.01, ***P < 0.001 compared with vehicle treatment. PKM2, pyruvate kinase M2; UUO, unilateral ureteral obstruction; FAN, folic acid nephropathy; HXK2, hexokinase 2; PDK1, pyruvate dehydrogenase kinase 1.

To validate whether our observations in cultured cells are also present in vivo, we used tandem mass spectrometry to analyze UUO and FAN mouse kidneys. This analysis revealed Cys358 modification of PKM2 and its inactivation (Supplemental Figure 3, A and B, and Supplemental Table 5). We also found that PKM2 exhibited a clear nuclear translocation pattern in kidney tubular epithelial cells after injury in both kidney injury models (Figure 7A). Furthermore, ALDH2-mutant mice showed higher expression levels of HIF-1α, PDK1, and HXK2 after injury compared with ALDH2 wild-type mice (Figure 7B). In diseased kidneys, we verified that the reduction in PKM2 activity varied by Aldh2 genotype, with Aldh2*2 homozygous mice showing the most significant decrease, followed by heterozygous mice, and wild-type mice showing the most minor reduction (Figure 7C). Conversely, the upregulation of HIF-1α signaling was the least in wild-type mice and the highest in Aldh2*2/*2 mice (Figure 7D). Additionally, mitochondrial ATP levels were markedly reduced in the kidneys of ALDH2-mutant mice across both fibrosis models (Figure 7, E and F).

### AAV-mediated ALDH2 overexpression restores tubular mitochondrial function and mitigates kidney fibrosis.

**Figure 8:**
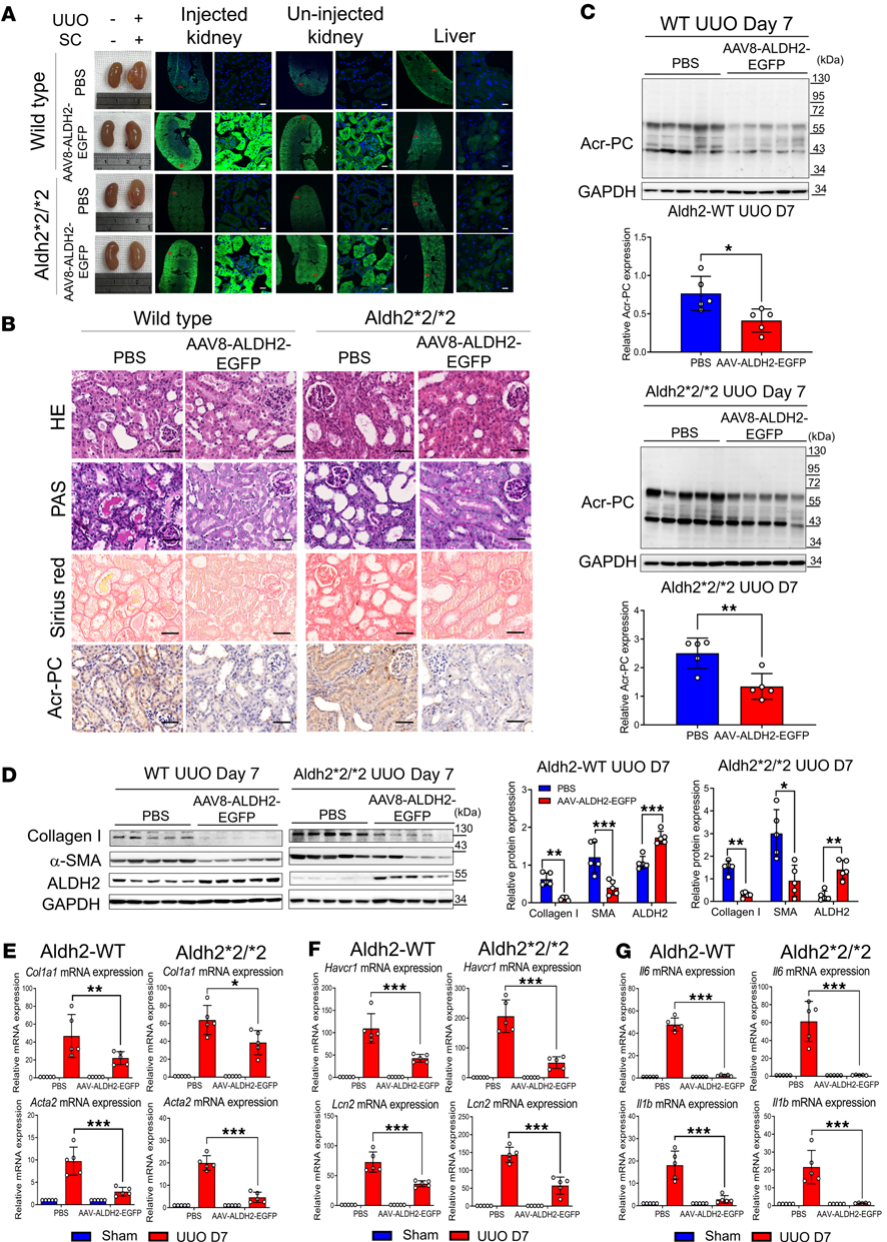
Impact of AAV-directed *ALDH2* gene on Acr-PC expression, fibrosis markers, inflammatory cytokines, and kidney damage markers in Aldh2*1 and Aldh2*2/*2 mice after UUO. Wild-type (WT) mice (*n* = 5) and Aldh2*2/*2 mice (*n* = 5) underwent subcapsular (SC) injections of 2 **×** 10^11^ genome copies of AAV8-ALDH2-EGFP for 28 days prior to UUO surgery. Mice were sacrificed 7 days after surgery. (**A**) Fluorescence images of ALDH2-EGFP in cryopreserved kidney and liver tissue sections were used to evaluate EGFP fluorescence signaling. Robust ALDH2-EGFP signaling was observed in the injected kidney, with some signal detected in the contralateral uninjected kidney. Low ALDH2-EGFP signaling was detected in the liver. Scale bar = 20 μm. (**B**) The first and second panels depict H&E and periodic acid–Schiff (PAS) staining, respectively, to assess morphological changes in kidney tissues. The third panel shows Sirius red staining to evaluate the kidney fibrosis area on day 7 after UUO. The fourth panel displays the immunohistochemistry of Acr-PCs in kidney tissues. Scale bar: 50 μm. (**C**) Western blot analysis of Acr-PCs in kidney tissues of mice is illustrated with quantification. (**D**) Western blot analysis of collagen 1, α–smooth muscle actin (α-SMA), and ALDH2 in kidney tissues of mice is illustrated with quantification. mRNA expression of (**E**) *Col1a1*, *Acta2*, (**F**) *Havcr1*, *Lcn2*, and (**G**) *Il6* and *Il1b* in kidney tissues was assessed through quantitative reverse transcription PCR analysis. Data are presented as mean ± SD. Statistical significance was determined using Mann-Whitney *U* or Kruskal-Wallis tests, and 2-tailed *P* values are indicated. *P < 0.05, **P < 0.01, ***P < 0.001 compared with the WT group. AAV, adeno-associated virus; *ALDH2*, aldehyde dehydrogenase 2; Acr-PCs, acrolein-protein conjugates; *Acta2*, actin alpha 2; *Col1a1*, collagen type I alpha 1 chain; *Havcr1*, hepatitis A virus cellular receptor 1 homolog; *Il6*, interleukin-6, *Il1b*, interleukin-1β; *Lcn2*, lipocalin-2.

To explore the potential of enhancing ALDH2 to prevent kidney injury, we used AAV8 delivery through subcapsular (SC) injections, bypassing the kidney size-exclusion barrier (25, 26). Following optimization of AAV8 dosage and delivery timing, validated by in vivo luminescence measurement, and verifying a comparable kidney injury response in mice through SC injection of both AAV8-EGFP vector and PBS (Supplemental Figure 4), we assessed the kidney-protective effects of AAV-mediated ALDH2 expression. Immunofluorescence images 28 days after SC injection revealed robust kidney transduction in wild-type and Aldh2*2/*2 mice (Figure 8A). Our findings indicate that AAV8-mediated ALDH2 overexpression effectively decreased Acr-PC accumulation, attenuated the inflammatory response, and mitigated kidney fibrosis severity in WT and ALDH2-mutant mice (Figure 8, B–G). ALDH2 overexpression also restored PK activity and ATP content and suppressed HIF-1α signaling in the UUO model (Supplemental Figure 5).

## Discussion

The present study provides insights into the role of ALDH2 in kidney fibrosis and its potential as a therapeutic target. Our results demonstrate that kidney ALDH2 expression level correlates with better kidney function, reduced IFTA, and a favorable long-term prognosis in CKD. In the animal experimental model, ALDH2 expression downregulates in the development of kidney fibrosis, accompanied by toxic Acr-PC accumulation, and our transgenic animal models showed that mice with decreased enzymatic activity because of an ALDH2 variationhave exacerbated Acr-PC accumulation and increased kidney fibrosis severity after kidney injury. Mechanistically, accumulated acrolein could trigger PKM2 nuclear translocation, which coactivates HIF-1α signaling and induces mitochondrial dysfunction. To address the potential clinical implication of our study, we showed that AAV-mediated ALDH2 delivery can mitigate Acr-PC accumulation and improve fibrosis. These findings highlight the role of ALDH2 in kidney fibrosis pathogenesis, offering insights into avenues for understanding and treating CKD.

Our study underscores the importance of ALDH2 in kidney health. We demonstrated that ALDH2 expression correlates with kidney fibrosis, urinary acrolein levels, and clinical outcomes in CKD. This study contributes to the field as it establishes a clear link between ALDH2 and kidney disease progression. It also suggests that ALDH2 could serve as a potential biomarker for kidney disease. This finding aligns with previous studies that have implicated ALDH2 in various kidney diseases (19, 27) and may also explain why individuals carrying the Glu504Lys polymorphism in ALDH2 have a higher risk of CKD (21). The precise mechanism behind ALDH2 downregulation in fibrotic kidneys is not yet fully understood, but it appears to involve multiple factors. Predominant oxidative stress (28) and inflammatory signaling pathways, such as JNK (29), contribute to this downregulation. Furthermore, dysregulation of critical pathways, including Wnt/β-catenin and TGF-β signaling, likely plays a crucial role in diminishing ALDH2 expression in fibrotic conditions (30, 31). These insights underscore the intricate regulatory network governing ALDH2 in the context of fibrotic disease. In contrast with the Aldh2-knockout mice, which have no ALDH2 enzyme activity and display age-related cognitive impairment and Alzheimer’s disease (32), mice carrying the Glu504Lys variant do not show any abnormalities in the nervous system and are more representative of clinical scenarios.

Earlier research has explored how ALDH2 confers protection to the kidneys during AKI by activating autophagy (17, 19), facilitating mitochondrial biogenesis via PGC-1α (18), and inhibiting the IκBα**/**NF-κB/IL-17C pathway (33). We showed that reduced ALDH2 enzyme activity results in acrolein accumulation in renal tubules, which induces PKM2 translocation to the nucleus and coactivates HIF-1α signaling. This finding not only deepens our understanding of the pathogenesis of kidney fibrosis but also identifies potential therapeutic targets. This is consistent with recent studies highlighting the role of PKM2 in kidney disease (34–36).

Our investigation presents compelling evidence regarding the therapeutic potential of ALDH2. We have demonstrated that AAV-mediated ALDH2 overexpression leads to a reduction of toxic aldehydes, restoration of tubular mitochondrial function, and attenuation of kidney fibrosis. Notably, our finding indicated that acrolein modifies PKM2 protein, prompting its translocation to the cell nucleus. This modification shifts mitochondrial oxidative phosphorylation toward aerobic glycolysis and activates HIF-1α transcription. Nuclear PKM2 then coactivates HIF-1α, forming a transcriptional complex that upregulates PDK1 and HXK2 expression in kidney tubular cells. These discoveries establish a clear association between acrolein-induced mitochondrial dysfunction and the progression of CKD.

In the realm of novel drug development, the efficacy of ALDH2 activators, such as AD-5591 and Alda-1, in mitigating tissue damage has been well documented across various organs (18, 19, 37–40). Recently, a novel and highly selective ALDH2 activator, AD-9308, has emerged, demonstrating promising outcomes in addressing diet-induced obesity and fatty liver and enhancing glucose homeostasis in both ALDH2 wild-type and mutation mice (41). In our study, we utilized AAV8-directed ALDH2 gene delivery to enhance ALDH2 activity, specifically within the kidneys. This intervention results in a substantial reduction in kidney damage, inflammation, and fibrosis. Notably, our analysis showed the highest ALDH2 expression in the kidney tubules, aligning with previous observations (26). To summarize, our findings underscore the therapeutic potential of modulating kidney ALDH2 expression as a strategy to attenuate the progression of kidney fibrosis.

Our study has limitations and highlights several areas that warrant further investigation. While we utilized kidney gene expression data and urine analysis to demonstrate the negative correlation between ALDH2 expression and kidney disease progression, we were unable to obtain ALDH2 genotyping of the study individuals because of IRB restrictions. Second, although we have established the crucial role of ALDH2 in kidney health, the precise mechanisms underlying its protective effects remain incompletely understood. Future studies should focus on elucidating these mechanisms to facilitate the development of more effective therapeutic strategies. Moreover, while our study provides compelling evidence for the therapeutic potential of ALDH2, further research is necessary to translate these findings into clinical practice.

In conclusion, our study reveals the role of ALDH2 in kidney fibrosis and its therapeutic potential. We established correlations between ALDH2 expression, kidney fibrosis, urinary acrolein levels, and CKD outcomes. Additionally, we elucidated molecular mechanisms underlying the involvement of ALDH2 in kidney fibrosis, providing compelling evidence for its therapeutic efficacy. Our findings address knowledge gaps and inspire future research, potentially leading to innovative therapeutic approaches for kidney disease.

## Methods

### Sex as a biological variable.

In our human studies, we examined men and women, and similar findings were reported for both sexes. In contrast, our animal studies used only male mice to avoid potential interference from sex hormones.

### Participants in the TRTOI study.

The Taipei Renal Transcriptomics and Outcomes Investigation (TRTOI) details were previously described (22); it is a prospective study conducted at Taipei Veterans General Hospital in Taiwan, focusing on adults undergoing native kidney biopsies or nephrectomies. TRTOI aims to analyze kidney transcriptomic profiles, seeking new biomarkers for patient outcomes. Kidney samples were processed using standardized techniques. An experienced nephropathologist, who remained unaware of ALDH2 results, performed histopathological analysis. Blood and urine samples were collected on the biopsy day.

### Estimation of GFR and assessment of chronic tubulointerstitial damage.

The Modification of Diet in Renal Disease formula estimated GFR. Chronic tubulointerstitial damage was evaluated in kidney biopsy samples using H&E, PAS, Jones’s Methenamine Silver, and Masson’s trichrome staining. The affected cortex area of IFTA was quantified for each stain and subsequently averaged to obtain an overall percentage.

### Gene expression analysis in the tubulointerstitial portion of TRTOI cohort patients.

A portion of the human kidney biopsy tissue was preserved in RNAlater (Invitrogen) and underwent manual microdissection to separate the glomerular and tubulointerstitial sections. The procedure for analyzing the mRNA expression pattern of the isolated tubulointerstitial component is described in the Supplemental Methods.

### MAKEs.

The primary outcome was a combination of MAKEs, which included a decrease in eGFR by 40% or greater from the baseline to the latest available measurement, kidney failure, or mortality due to cardiovascular or kidney-related causes. Participants were followed until kidney failure, death, loss to follow-up, or study completion (January 31, 2023), with no loss to follow-up reported.

### Analysis of urinary acrolein levels.

Urinary acrolein levels (acrolein/creatinine) were measured using a previously described competitive enzyme immunoassay (14).

### Animal experiments.

Mice were individually housed under controlled conditions of temperature (23°C) on a 12-hour light/12-hour dark cycle, with unrestricted access to food and water. This study utilized male C57BL/6 mice, the wild-type mice, and Aldh2*2 E487K-knockin mutant mice, reflecting the East Asian E487K mutation (Aldh2*2 mice), aged 6–10 weeks. We exclusively selected male mice for the experiment to mitigate any potential interference from sex hormones. The Aldh2*2 mice, developed by Daria Mochly-Rosen (42), were bred and raised in-house, with genotyping performed by Genomics Inc. For the folic acid–induced interstitial fibrosis model, male C57BL/6 mice with Aldh2 wild-type or mutant Aldh2 homozygous (Aldh2*2/*2) and heterozygous genotypes (Aldh2*1/*2), aged 8 to 10 weeks, were intraperitoneally injected with a single dose of 225 mg/kg folic acid (MilliporeSigma) in 0.3 mM NaHCO_3_ solution. They were sacrificed at 28 days, as previously outlined (43, 44). In the UUO model, male C57BL/6 mice with Aldh2 wild-type or Aldh2*2/*2 and Aldh2*1/*2 genotype, aged 6 to 8 weeks, were anesthetized using 1%–3% isoflurane. The left kidney’s proximal ureter was ligated with 6–0 suture silk through a subcostal lateral incision. Sham-operated mice underwent the same surgical procedure without ligation. Following the surgical procedure, mice were initially placed in individual housing and subsequently transitioned to communal housing under standard conditions for either 7 or 14 days after surgery.

### Intrarenal delivery of ALDH2 gene using AAV8 viral vector.

Mice for the ALDH2 gene or PBS administration were chosen randomly. Under 1%–3% isoflurane anesthesia, 20 μL/site of AAV8 (containing 10^12^ viral particles, diluted in PBS) with AAV8-ALDH2-EGFP construct (see Supplemental Figure 6) was gently injected into the renal capsule using an insulin syringe. To maintain kidney moisture, warm sterile saline was applied before AAV delivery. The needle of the AAV-containing syringe pierced the kidney capsule’s upper end and then moved slowly along the inner surface from upper to lower. At the needle’s midpoint, AAV was injected gradually; afterward, the syringe was withdrawn slowly, and the injection site was compressed using a cotton swab for 3–5 minutes. Once the matrix solidified, the kidney was returned to the abdominal cavity, and the wound was closed with stitches. The animals were kept on a heating pad until recovery and returned to animal facilities for monitoring. Additionally, a control group received intrarenal PBS injections.

### Biochemical analyses.

Before sacrifice, the blood of these animals was collected from the submandibular (facial) vein for indicated time points. After sacrifice, blood samples were collected by cardiac puncture using heparinized syringes. Serum was collected after centrifugation at 12,000 rpm for 5 minutes. Plasma levels of uric acid, blood urea nitrogen, and creatinine were assessed utilizing the FUJI DRI-CHEM 4000i Automated Clinical Chemistry Analyzer.

### Histological, IHC, and immunofluorescence staining analyses.

Tissues were fixed using 4% paraformaldehyde for 24 hours, followed by paraffin embedding for subsequent histology, IHC analyses, and immunofluorescence staining, as described in the Supplemental Methods.

### Mitochondrial isolation.

After euthanizing the mice, mitochondrial isolation from fresh kidney tissue followed previously described procedures (45) (see Supplemental Methods).

### ALDH2 activity and PK activity assays.

The ALDH2 activity and PK activity in tissues or cells were evaluated using the Mitochondrial ALDH2 Activity Assay kit (Abcam, ab115348) and PK Assay Kit (Abcam, ab83432), respectively, following the manufacturer’s instructions as described in the Supplemental Methods.

### Cell culture.

The information regarding cell line and primary mouse renal tubule epithelial cell culture is described in the Supplemental Methods.

### Intracellular and mitochondrial ROS analysis.

To assess cytosolic and mitochondrial ROS generation, dichlorofluorescein and MitoSox staining assays were utilized as described in Supplemental Methods.

### Detection of mitochondrial membrane potential and ATP assays.

The mitochondrial membrane potential was evaluated using JC-1, and the procedure for ATP analysis is detailed in Supplemental Methods.

### Mitochondrial respiration analysis and Real-Time ATP Rate Assay.

Mitochondrial respiration analysis and Real-Time ATP Rate Assay were conducted using the Seahorse XF24 Extracellular Flux Analyzer (Seahorse Bioscience), following the manufacturer’s instructions outlined in Supplemental Methods.

### Quantitative real-time RT-PCR.

The procedure for total RNA extraction, reverse transcription, and real-time RT-PCR analysis is described in Supplemental Methods.

### Cell fractionation and immunoblotting analysis.

Cell fractionation and immunoblotting analysis was performed following the procedure for preparing and evaluating cell lysates described in previously reported methods (46), outlined in the Supplemental Methods.

### ChIP assay.

The ChIP and real-time PCR quantification were conducted as described in established procedures, outlined in Supplemental Methods.

### Protein modification analysis by in-gel digestion and liquid chromatography-tandem mass spectrometry.

In-gel digestion and protein modification identification were conducted following established protocols, as described in the Supplemental Methods.

### Statistics.

Data are presented as means with SD or as numbers with percentages. The relationships between kidney *ALDH2* mRNA levels and variables of interest were examined, and the respective Spearman’s rank correlation coefficients were presented. To explore the association between kidney *ALDH2* mRNA levels and MAKEs, participants were grouped based on *ALDH2* tertile levels. Kaplan-Meier analysis, with *ALDH2* as the grouping factor, assessed cumulative MAKE risk, and comparisons were made using the log-rank test. We also performed multivariable Cox proportional regression analysis to ascertain the independent association of kidney *ALDH2* mRNA with MAKEs, initially without adjustments and subsequently adjusting for age, sex, proteinuria, the presence of DKD, and eGFR, ensuring no significant collinearity in the multivariable analysis. Significance was assessed using Mann-Whitney *U* or Kruskal-Wallis tests with 2-tailed *P* values. All statistical computations were conducted using SPSS (version 23) and GraphPad Prism 9 (GraphPad Software), with a significance threshold set at *P* < 0.05.

### Study approval.

The study design of human studies was approved by the Institutional Review Board of Taipei Veterans General Hospital, adhering to Helsinki Declaration principles (IRB2021-08-013B). Eligible patients provided written informed consent. All animal experiments were conducted following approval by the Institutional Animal Care and Use Committee of National Yang Ming Chiao Tung University, adhering to the Guidelines for Animal Research of the institution (IACUC 1110808r).

### Data availability.

Supporting Data Values associated with the main manuscript and supplement material are provided in the Supporting Data Values file.

## Author contributions

SYL, MTT, CCL, and HTW designed the study, performed research, and analyzed data. YMK, HMY, ZJT, and HWC performed the experiments and analyzed the data. MTT, SYL, and CCL collected clinical samples and analyzed clinical data. SYL, MTT, and HTW wrote the paper.

## Supplementary Material

Supplemental data

Unedited blot and gel images

Supporting data values

## Version 1 09/03/2024

In-Press Preview

## Version 2 10/08/2024

Electronic publication
