# Supplementary material for: Aldehyde dehydrogenase 2 preserves kidney function by countering acrolein-induced metabolic and mitochondrial dysfunction
Source: JCI Insight. 2024 Oct 8;9(19):e179871. doi: 10.1172/jci.insight.179871 (PMC11466184; doi:10.1172/jci.insight.179871)
Supplement: Supplemental data [file jciinsight-9-179871-s020.pdf]

Aldehyde dehydrogenase 2 preserves kidney function by countering acrolein-induced metabolic and mitochondrial dysfunction.

Szu-yuan Li[1,2#], Ming-Tsun Tsai[1,2#], Yu-Ming Kuo[3], Hui-Min Yang[3], Zhen-Jie Tong[3], Hsiao-Wei Cheng[3], Chih-Ching Lin[1,2]*, Hsiang-Tsui Wang[3,4,5]*

[1]Division of Nephrology, Department of Medicine, Taipei Veterans General Hospital, Taipei, Taiwan, ROC.
[2]School of Medicine, College of Medicine, National Yang Ming Chiao Tung University, Taipei, Taiwan, ROC.
[3]Institute of Pharmacology, College of Medicine, National Yang Ming Chiao Tung University, Taipei, Taiwan, ROC.
[4]Institute of Food Safety and Health Risk Assessment, National Yang Ming Chiao Tung University, Taipei, Taiwan, ROC.
[5]Doctor degree program in Toxicology, Kaohsiung Medical University, Kaohsiung, Taiwan, ROC.
*Co-corresponding author
[#] These authors contributed equally to this work.
Address: No.155, Sec.2, Linong Street, Taipei, 112 Taiwan (ROC)
E-mail: htwang01@nycu.edu.tw (H-T Wang); lincc2@vghtpe.gov.tw (C-C L)
Tel: 886-02-2826-7097

Supplementary methods

Reagents and kits. Acrolein (110221), collagen I (C3867), collagenase (C5138), 2',7'-dichlorofluorescin diacetate (DCFH-DA), Anti-HIF-1α antibody (ABE279), JC-1 dye (T4069), Hematoxylin-Eosin Staining Solution (A3429), hydrocortisone (H0888), and paraformaldehyde (158127), Periodic Acid-Schiff (PAS) Staining System (395B) were purchased from Sigma (St. Louis, MO, USA). ATP bioluminescence assay kit HS II (11699709001) was purchased from Roche (Boston, USA). MitoSOX™ mitochondrial superoxide indicators and TRIzol reagent were purchased from Invitrogen (Carlsbad, USA). The Agilent Seahorse XF Cell Mito Stress Test and Seahorse XF Real-Time ATP Rate Assay Kit were purchased from Agilent Technologies (Santa Clara, CA, USA). The KAPA SYBR Fast Master Mix ABI Prism qPCR kit was purchased from KAPA Biosystems (Wilmington, DE, USA). Hank's balanced salt solution (HBSS, 14025092), insulin/ transferrin/ selenium (ITS, 41400045), Recombinant human epidermal growth factor (rhEGF, PHG0311L) and RevertAid First Strand cDNA Synthesis Kit were purchased from Thermo Scientific (Waltham, MA, USA). The Cell Fractionation Kit (#9030) and primary antibodies against α-SMA (#19245), GAPDH (#5174), HXK2 (#2867), Lamin B1 (#12586), PKM2 (#4053), VDAC (#4661), and Vimentin (#5741) were purchased from Cell Signaling Technology (Beverly, MA, USA). ALDH2 (Abcam, ab227021), collagen I (1:1000, Abcam, ab6308), 4-hydroxynonenal antibody (4-HNE, ab46545), Mitochondrial

ALDH2 Activity Assay kit (ab115348), PK Assay Kit (ab83432), and Picro-Sirius Red Solution (ab246832) were purchased from Abcam (Boston, MA, USA). Goat anti-rabbit-IgG (AP132P), goat-anti-mouse-IgG (AP124P), and enhanced chemiluminescence (ECL) reagents were purchased from Millipore (Burlington, VT, USA). Dulbecco's Modified Eagle's Medium (DMEM), DMEM/F-12 (Dulbecco's Modified Eagle Medium/Nutrient Mixture F-12), horse serum, and fetal bovine serum (FBS) were purchased from Life Technologies Corporation (Grand Island, NY, USA). Antibiotic-antimycotic agents and trypsin-EDTA solution for cell culture were purchased from Biological Industries (Foreston, MN, USA).

Histological and immunohistochemical analysis. Tissues were fixed using 4% paraformaldehyde for 24 hours, followed by paraffin embedding for subsequent histology and immunohistochemistry analyses. Once paraffin-embedded, each tissue sample was sectioned into four cross-sections, which were then mounted onto slides. Standard hematoxylin and eosin (H&E) staining was carried out, while Sirius red staining assays were employed to assess renal fibrosis. To evaluate renal structural changes, hemisected kidneys preserved in formalin were subjected to Periodic Acid Schiff (PAS) staining. Histological assessments were conducted by two independent, blinded observers—an anatomical pathologist and a nephrologist—using a light microscope, as previously outlined (1, 2). For immunohistochemical staining or immunofluorescent staining assays, antigen retrieval was achieved by heating the sections on slides with citraconic anhydride, followed by incubation with primary antibodies.

Immunofluorescent staining assay. The procedure of immunofluorescence assay was described previously (3, 4). For PKM2 translocation analysis, acrolein-treated cells were seeded, fixed, and permeabilization with 0.5% (v/v) Triton X-100. After blocking in 0.3% (w/v) BSA and 0.05% (v/v) Tween-20 in PBS at room temperature for 1 hour, cells or tissue sections with antigen retrieval were incubated with PKM2 (1:200, Cell Signaling) at 4°C for overnight, followed by incubation of 80 ng/mL DAPI at room temperature for 1 hour. The suitable fluorophore-conjugated secondary antibodies (1:200, FITC; Molecular Probes) were utilized, and immunofluorescent images were observed with a fluorescence laser-scanning confocal microscope (Olympus FV10i).

***In vivo* bioluminescence imaging.** The experiment involved anesthetizing mice with isoflurane and then administering a 150 μL injection of D-Luciferin (15 mg/mL; Cayman) intraperitoneally. Subsequently, images were captured using the IVIS® Lumina X5 Imaging System from Perkinelmer, Inc., approximately ten minutes after the administration of D-Luciferin. Luminescence measurements were obtained and analyzed using Living Image software.

Mitochondrial isolation. Immediately following euthanasia of the mice, mitochondrial isolation from fresh kidney tissue was conducted in accordance with previously described procedures (5). All steps were carried out using prechilled equipment and solutions. In brief, kidney tissues were

harvested from the mice and rinsed with PBS. For mitochondrial isolation, the tissue was weighed and immersed in an isolation buffer (comprising 210 mM mannitol, 70 mM sucrose, 5 mM HEPES, 1 mM EGTA, and 0.2% (w/v) fatty acid-free BSA, pH 7.2) at a ratio of approximately 100 mg of tissue per 1 mL of buffer. The tissues were then homogenized with ten strokes using a Dounce homogenizer, maintaining the tube's temperature on ice throughout the process. This was followed by differential centrifugation, as previously outlined. The resulting pellets were subsequently reconstituted in BSA-free isolation buffer, and protein concentration was determined using a BCA assay (Thermo).

Aldehyde dehydrogenase 2 (ALDH2) activity assay. The ALDH2 activity in tissues was evaluated using the Mitochondrial Aldehyde Dehydrogenase (ALDH2) Activity Assay kit (ab115348) as per the manufacturer's instructions. To begin, tissue lysates were prepared using the Extraction buffer, followed by centrifugation and quantification through a BCA protein assay. Subsequently, an equivalent amount of protein lysates (100 µL) was introduced into microplates coated with ALDH2 antibody and then allowed to incubate for 3 hours at room temperature. After thorough washing using 1x Wash buffer, 200 µL of 1x Activity solution was added, and the ALDH2 activity was gauged using a Multimode microplate reader (TECAN, Infinite 200) by measuring the increase in absorbance at 450 nm resulting from the NADH formation. The ALDH2 activity was expressed as the alteration in absorbance per minute per quantity of sample loaded into the well.

Pyruvate kinase (PK) activity assay. Pyruvate kinase activity was determined using the Pyruvate Kinase Assay Kit (Abcam, ab83432), following the manufacturer's instructions. For the analysis of PK activity, we used kidney lysates (50µg), cell lysates obtained from acrolein-treated HK2 cells (50µg), or 5µL of acrolein-treated pyruvate kinase (Roche; equivalent to 0.5mU). The relative PK activity was quantified based on a pyruvate standard curve and expressed in mU/mL relative to a control.

Primary mouse renal tubular epithelial cell culture. Mouse primary proximal tubules were isolated following established procedures as previously described (6). Briefly, mice were anesthetized with Isoflurane and euthanized by cervical dislocation. Kidneys were promptly excised and placed in cold Hanks Balanced Salt Solution (HBSS, Thermo Scientific) supplemented with 1% antibiotic/antimycotic (Biological Industries). After removing the renal capsule, each kidney was halved sagittally, and the medulla was discarded. The remaining cortical tissue was minced and treated with Collagenase (1 mg/mL, Sigma) in HBSS for 30 minutes at 37°C while rotating. Following digestion, tubular cells were harvested using a series of cell strainers and resuspended in DMEM/F-12 culture media (Thermo Scientific) containing insulin/ transferrin/ selenium (Thermo Scientific), 0.2 µM hydrocortisone (Sigma-Aldrich), 0.01 mg/mL recombinant human epidermal growth factor (rhEGF, Thermo Scientific).

NRK-52E Cell culture and acrolein treatment. The NRK-52E rat proximal tubular epithelial cell line (BCRC#60086) obtained from the Bioresource Collection and Research Center was maintained in Dulbecco's modified Eagle's medium supplemented with 10% heat-inactivated FBS. Before treatment, a freshly prepared stock solution of acrolein (Sigma) was employed for various doses of acrolein treatment (0-30 μM) for 1 hour or 24 hours.

Cell fractionation. Both cytoplasmic and nuclear fractions were prepared from cells using the cell fractionation kit (Cell Signaling, #9038) following the manufacturer's instructions. The cytoplasmic fraction was separated by straightforward centrifugation at 800 g for 15 minutes on cell lysates, while the nuclear fraction consisted of the cell pellets post-centrifugation, which were subsequently collected for further nuclear lysis.

Intracellular and mitochondrial ROS analysis. To assess cytosolic and mitochondrial ROS generation, DCF (dichlorofluorescein) and MitoSox staining assays were utilized. NRK-52E cells were exposed to acrolein (0-30 μM) for 1 hour, followed by treatment with a 5- (and-6)-chloromethyl-2′,7′-dichlorodihydrofluorescein diacetate (CM-H2DCFDA) probe (Sigma) at 10 μM in PBS (0.5 mL/well or insert) for 30 minutes at 37 °C. For mitochondrial ROS detection, MitoSox red (5 μM, Molecular probe, M36008) and acrolein (0-30 μM) were applied to NRK-52E cells for 30 minutes, adhering to the manufacturer's instructions. Flow cytometry (FACSCalibur, BD) was employed to quantify intracellular and mitochondrial ROS levels.

Detection of mitochondrial membrane potential. The mitochondrial membrane potential was evaluated using JC-1(7). NRK-52E cells underwent a 1-hour treatment with acrolein (0-30 μM) or carbonyl cyanide m-chlorophenyl hydrazone (CCCP, 100 μM, Sigma) as a positive control. After treatment, the cells were collected and suspended in JC-1 dye (Molecular Probe, T3168) (5 μM) in PBS for 30 minutes at room temperature in the dark, followed by flow cytometry using FACS Calibur (BD).

Adenosine triphosphate (ATP) analysis. NRK-52E cells were subjected to a 1-hour treatment with acrolein (0-30 μM) or CCCP (100 μM, Sigma) as a positive control. Subsequently, the cells were collected, lysed using cell lysis reagent, and the ATP content in the cell lysates quantified using the ATP bioluminescence assay kit HS II (Roche Molecular Biochemicals) with a Multimode microplate reader (TECAN, Infinite 200).

Mitochondrial respiration analysis and real-time ATP rate assay. Mitochondrial respiration and ATP production rates from both mitochondria and glycolysis were assessed using the Seahorse XF24 Extracellular Flux Analyzer (Seahorse Bioscience, North Billerica, MA). The Cell Mito Stress Test and Real-Time ATP Rate Assay Kit (Agilent Technologies) were employed for these analyses following the manufacturer's protocols. In brief, NRK-52E cells or primary mouse renal tubule

epithelial cells were cultured on Seahorse XF-24 plates and treated with acrolein (0-30 μM) for 1 hour. Group data of primary mouse renal tubule epithelial cells in Figure 6J from original tracings illustrate OCR changes in response to sequential administration of oligomycin (1 μM), carbonyl cyanide 4-(trifluoromethoxy)phenylhydrazone (FCCP; 1 μM), and rotenone/antimycin A (1 μM). The plates were retained for protein analysis following the experiments to ensure equivalent cell numbers. Respiration rates were determined as the mean ± SEM from four independent experiments, each conducted with three replicate wells using the Seahorse XFe24 analyzer.

Quantitative real-time RT-PCR. Total RNA extraction was performed using TRIzol® Reagent (Thermo Fisher Scientific). Subsequently, reverse transcription was carried out using RevertAid Reverse Transcriptase (Thermo Fisher Scientific) as per the manufacturer's protocol. The resulting cDNA was subjected to real-time RT-PCR analysis in triplicate using the StepOnePlus™ Real-Time PCR System (Applied Biosystems) with SYBR green dye. Primer sequences for the quantitative real-time PCR assay are provided below. Primers (5′-3′) were CTTCGTGACTACTGCCGAG and ATAGGTGGTTTCGTGGATGC for *Acta2*; CCCAATGGTGAGACGTGGAA and TTGGGTCCCTCGACTCCTAC for *Col1a1*; CTGGAATGGCACTGTGACATCC and GCAGATGCCAACATAGAAGCCC for *Havcr1*; ATGTCACCTCCATCCTGGTCAG and GCCACTTGCACATTGTAGCTCTG for *Lcn2*; ACAACCACGGCCTTCCCTACTT and CACGATTTCCCAGAGAACATGTG for *Il6*; TGCAGCTGGAGAGTGTGGATCCC and TGTGCTCTGCTTGTGAGGTGCTG for *Il1b*; GGAGGAACCTGCCAAGTATG and TGGGAGTTGCTGTTGAAG for GAPDH. GAPDH was employed as an internal control for all qRT-PCR experiments, and its expression was compared to that of control groups to determine the relative RNA expression.

Cell fractionation. Cytosolic and nuclear fractions of NRK-52E were obtained using a Cell Fractionation Kit (Cell signaling, #9030) following the manufacturer's protocol. Initially, cells were harvested and rinsed with PBS. Subsequently, they were homogenized in cytoplasm isolation buffer at 4 °C for 5 minutes, followed by centrifugation at 500 × g for 5 minutes to collect the cytosolic fraction. The resulting supernatant was retained. The pellet was then resuspended in membrane isolation buffer at 4 °C for 5 minutes and subjected to centrifugation at 8,000 × g for 5 minutes to obtain the nuclear fraction, which was collected from the pellet.

Immunoblotting analysis. The procedure for preparing and evaluating cell lysates was carried out in accordance with previously described methods (8). In brief, following a blocking step with 5% nonfat milk, the blots were incubated overnight at 4 °C with primary antibodies. The immunoblotting utilized the following antibodies: α-SMA (1:1000, Cell Signaling #19245), ALDH2 (Abcam, ab227021), Acr-PC antibody (1:1000, developed in-house) (9), collagen I (1:1000, Abcam, ab6308), GAPDH (1:5000, Cell Signaling #5174), HIF-1α (1:1000, Sigma # ABE279), HXK2 (1:1000, Cell Signaling #2867), 4-hydroxynonenal antibody (4-HNE, 1:1000, Abcam, ab46545), Lamin B1

(1:1000, Cell Signaling #12586), PKM2 (1:1000, Cell Signaling #4053), VDAC (1:1000, Cell Signaling #4661), and Vimentin (1:1000, Cell Signaling #5741). The detection of immunoreactive bands was accomplished using enhanced chemiluminescence (ECL) (Millipore Corporation, Billerica, MA, USA).

Chromatin immunoprecipitation (ChIP) assay. The chromatin immunoprecipitation and real-time PCR quantification were conducted following established procedures (10, 11). In brief, NRK-52E cells treated with acrolein (20 μM, 24h) underwent formaldehyde cross-linking. Fragmented chromatin was immunoprecipitated using anti-HIF-1 alpha (Abcam, ab179483) and anti-PKM2 antibodies (Cell Signaling, #4053). Rabbit immunoglobulin G (IgG) antibody (Cell Signaling) was a control. The total input consisted of the supernatant from the no-antibody control. Primer sequences for the real-time PCR assay are provided below. Primers (5'-3') were TCCCCTGTGTCTTAGGTGCT and GCCCCCTGCTTTGTTTTT for PDK1; GATCGCCTGCTTATTCACGG and CGGAGAGCACGCAACTTG for HXK2.

In-gel digestion and protein modification through liquid chromatography-tandem mass spectrometry (LC-MS/MS) analysis. The method for in-gel digestion and protein identification was conducted following established protocols (12). In summary, proteins (50 μg) were separated using 8% SDS-PAGE, and bands were excised based on the molecular weight of PKM2. Following destaining and SpeedVic-assisted drying of gel pieces, the dried gel portions were rehydrated with trypsin in 25 mM NH_4HCO_3, then incubated at 37°C overnight. Supernatants were collected, and the remaining peptides were extracted sequentially using 25 mM NH_4HCO_3 and 25 mM NH_4HCO_3/50% acetonitrile (ACN). After collecting and drying the supernatants, the dried digests were stored at -20°C for later use. The protein digests were analyzed using an Agilent 1200 nanoflow HPLC system (Agilent Technologies, Santa Clara, CA, USA) connected to an LTQ-Orbitrap hybrid tandem mass spectrometer (ThermoFisher, Waltham, MA, USA). All MS/MS data were subsequently employed for searches utilizing the TurboSEQUEST program (Thermo Electron) with the UniProt protein sequence database for mouse proteins.

Supplementary Tables

Supplementary Table 1. Baseline characteristics of participants in this study (*N* = 81).

Parameters	
Age, yr	54.7 ± 16.7
Male, *n* (%)	42 (51.9)
eGFR, mL/min/1.73 m^2	51 (20−91)
Proteinuria, mg/mg creatinine	2.3 (1.1−7.7)
Clinicopathologic diagnostic categories, *n* (%)	
Proliferative glomerulonephritis	22 (27.2)
Nonproliferative glomerulopathies	34 (42.0)
Diabetic nephropathy	8 (9.9)
Vascular	8 (9.9)
Tubulointerstitial	4 (4.9)
Other	5 (6.2)
Medications, *n* (%)	
ACEi/ARB	41 (50.6)
Beta-blockers	29 (35.8)
Calcium channel blockers	41 (50.6)
Glucocorticoids	52 (64.2)
Immunosuppressants other than glucocorticoids	14 (17.3)

Data are presented as mean ± standard deviation, median [interquartile range], and count with percentages. Abbreviations: ACEi, angiotensin-converting enzyme inhibitors; ARB, angiotensin II type 1 receptor blockers; eGFR, estimated glomerular filtration rate.

Supplementary Table 2. Renal function, proteinuria, and *ALDH2* expression levels by primary clinicopathologic diagnosis.

	PGN	NPGP	DKD	Vascular	TI	Other	*p*
eGFR, ml/min/1.73m^2	56 (24−98)	77 (42−98)	22 (11−58)	20 (16−38)	21 (8−34)	27 (5−108)	0.002
Proteinuria, mg/mg Cre	1.5 (0.4−2.5)	6.2 (2.4−11.0)	4.0 (1.9−11.4)	1.3 (0.4−3.8)	0.3 (0.2−0.4)	1.0 (0.2−3.1)	<0.001
ALDH2 expression (TPM)	330 (273−397)	400 (346−429)	237 (201−358)	269 (245−362)	278 (171−349)	215 (148−520)	0.001

Data presented as median [interquartile range]. Abbreviations: Cre, creatinine; DKD, diabetic kidney disease; eGFR, estimated glomerular filtration rate; NPGN, nonproliferative glomerulopathies; PGN, proliferative glomerulonephritis; TI, tubulointerstitial; *ALDH2*, aldehyde dehydrogenase 2.

Supplementary Table 3. Associations between kidney ALDH2 mRNA levels and major adverse kidney events

Variables	HR (95%CI)	HR (95%CI)	HR (95%CI)
Kidney *ALDH2* mRNA			
Per 1-unit increase in Log2 (TPM)	0.34 (0.19−0.61)	0.34 (0.18−0.65)	0.96 (0.39−2.35)

Model 1 is unadjusted.
Model 2 is adjusted for age, sex, proteinuria, and presence of diabetic kidney disease.
Model 3 includes the adjustments made in Model 2 and is further adjusted for baseline eGFR.
Abbreviations: *ALDH2*, aldehyde dehydrogenase 2; CI, confidence interval; eGFR, estimated glomerular filtration rate; HR, hazard ratio; TPM, transcript per million.

Supplementary Table 4. Acrolein-modified PKM2 residue in NRK-52E treated with acrolein.

Z	$[M+H]^{+}_{obs}$	$[M+H]^{+}_{cal}$	△m (ppm)	Modified Residues	Modified peptide
3+	3508.640	3508.649	-2.83	Cys358	^{342}AEGSDVANAVLDGAD$_{Acr}$CIM*LSGETAKGDYPLEAVR^{376}
2+	2508.156	2508.144	-4.67	Cys358	^{342}AEGSDVANAVLDGAD$_{Acr}$CIM*LSGETAK^{367}

Z represents charge state, and $[M+H]^{+}_{obs}$ and $[M+H]^{+}_{cal}$ represent observed and calculated mass. The △m indicates the error calculated by observed $[M+H]^{+}$ minus calculated $[M+H]^{+}$ and the results divided by calculated $[M+H]^{+}$, ppm means parts per million. Modified peptide sequences were listed as well. Acr represents acrolein modification with mass changes of 56.0262 Da. Abbreviation: PKM2: pyruvate kinase M2.

Supplementary Table 5. Acrolein-modified PKM2 residue in kidney tissues of mice through UUO or FAN.

Z	$[M+H]^+_{obs}$	$[M+H]^+_{cal}$	△m (ppm)	Modified Residues	Modified peptide
UUO					
3+	3508.655	3508.649	-1.57	Cys358	^{342}AEGSDVANAVLDGAD$_{Acr}$CIM*LSGETAKGDYPLEAVR^{376}
3+	2508.154	2508.144	-3.96	Cys358	^{342}AEGSDVANAVLDGAD$_{Acr}$CIM*LSGETAK^{367}
FAN					
3+	3508.650	3508.649	-0.20	Cys358	^{342}AEGSDVANAVLDGAD$_{Acr}$CIM*LSGETAKGDYPLEAVR^{376}
3+	2508.156	2508.144	-4.75	Cys358	^{342}AEGSDVANAVLDGAD$_{Acr}$CIM*LSGETAK^{367}

Z represents charge state, and $[M+H]^+_{obs}$ and $[M+H]^+_{cal}$ represent observed and calculated mass. The △m indicates the error calculated by observed $[M+H]^+$ minus calculated $[M+H]^+$ and the results divided by calculated $[M+H]^+$, ppm means parts per million. Modified peptide sequences were listed as well. Acr represents acrolein modification with mass changes of 56.0262 Da. Abbreviations: PKM2: pyruvate kinase M2; UUO: unilateral ureteral obstruction; FAN: folic acid nephropathy.

Supplementary Figures

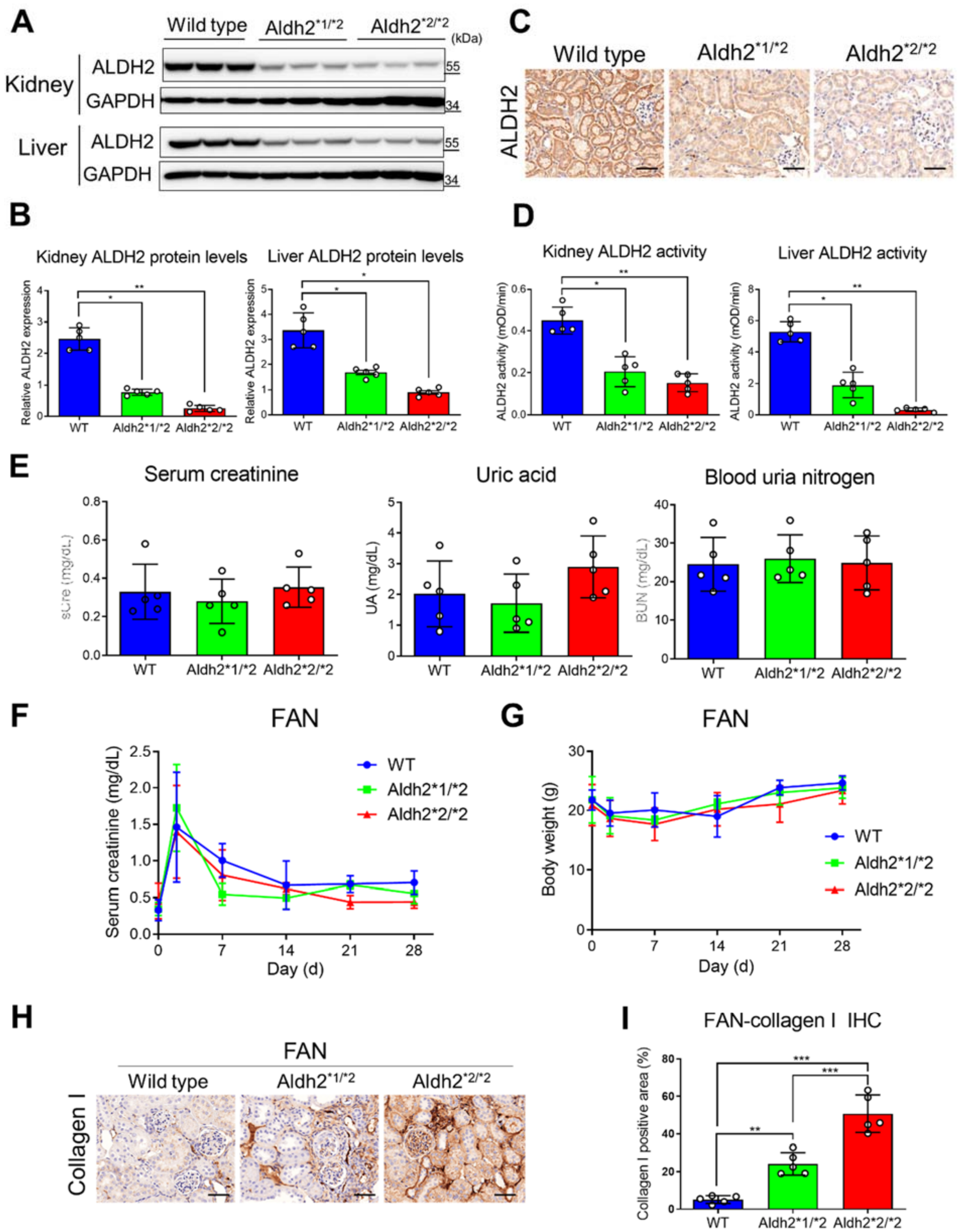

Supplementary Figure 1. Protein expression and enzymatic activity of ALDH2 in kidney and liver tissues of mice with different Aldh2 genotypes. (A) ALDH2 expression in kidney and liver tissues of Aldh2*1 (wild type), Aldh2*1/*2 (heterozygotes), and Aldh2*2/*2 (homozygotes) mice

was assessed using Western blot analysis. (B) Quantification of the results from A. (C) Immunohistochemical (IHC) staining of ALDH2 in kidney tissues of the various mice groups, with a scale bar of 50 μm. (D) ALDH2 activity in different mice groups was measured through the Mitochondrial Aldehyde Dehydrogenase (ALDH2) Activity Assay Kit (Abcam, ab115348). Kidney and liver tissue lysates were diluted to 40 and 10 mg/mL, respectively. (E) Serum creatinine, blood uric acid, and blood urea nitrogen levels were assessed in various groups of mice. (F-G) Aldh2*1 (wild type), Aldh2*1/*2, and Aldh2*2/*2 mice (n=5 for each group) were intraperitoneally administered folic acid (FA, 225 mg/kg in 300 mM $NaHCO_3$) and then sacrificed on day 28. (F) Measurement of serum creatinine and (G) body weight of mice on days 0, 3, 7, 14, 21, and 28 after FA injection. (H) IHC staining of collagen I in kidney tissues of Aldh2 wild type, Aldh2*1/*2, and Aldh2*2/*2 mice exposed to FA injection day 28 as described in **Figure 5B**, with a scale bar of 50 μm. (I) The quantification of collagen I positive area of H. Data are presented as mean ± SD. Statistical significance was determined using Kruskal-Wallis tests, and two-tailed p values are indicated. *$p<0.05$, **$p<0.01$ compared to the wild-type (WT) group. Abbreviations: ALDH2: aldehyde dehydrogenase 2; FA: folic acid; FAN: folic acid nephropathy; IHC: immunohistochemical; WT: wild-type.

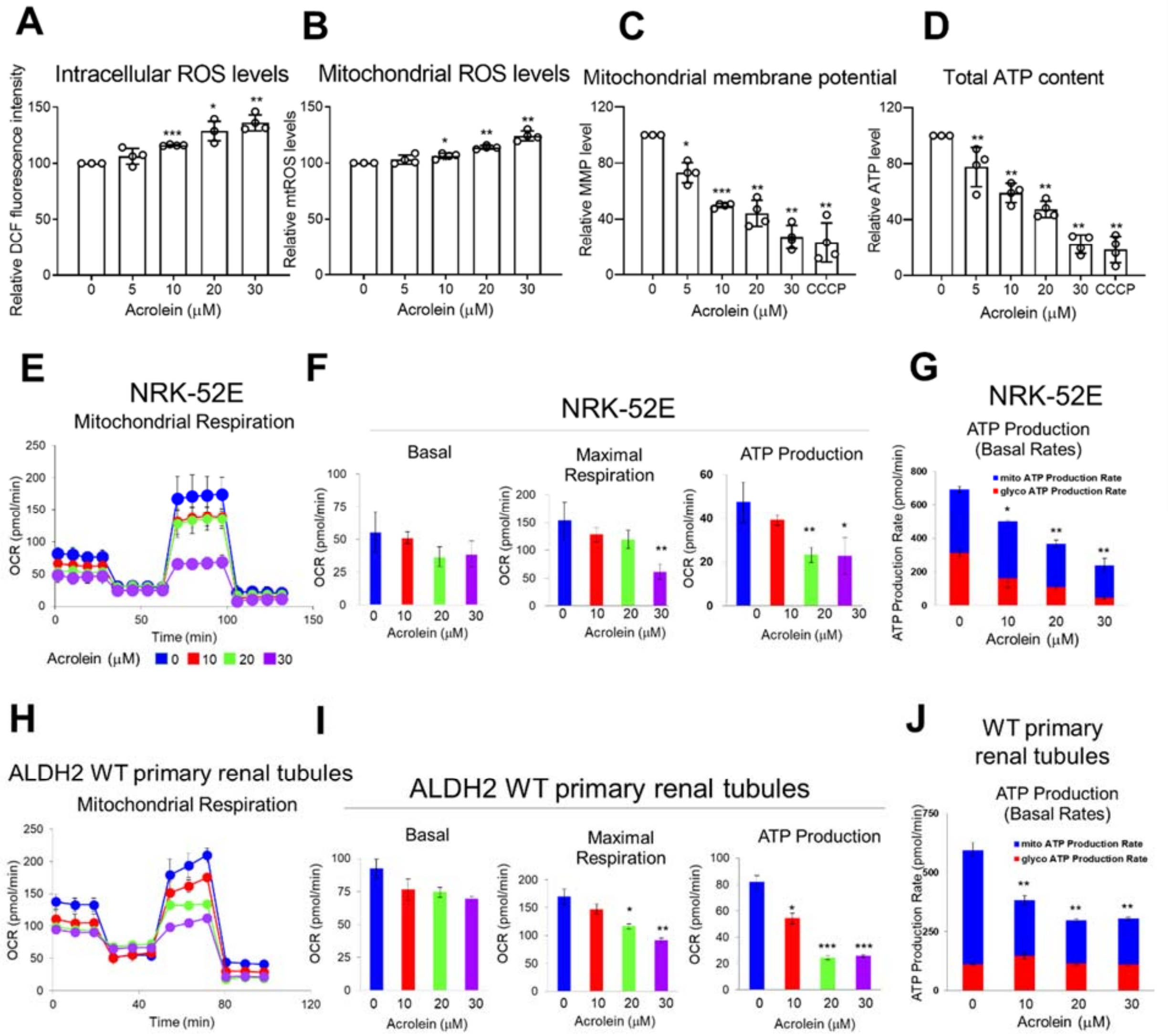

Supplementary Figure 2. The effect of acrolein on mitochondrial function in NRK-52E cells and primary mouse renal tubular epithelial cells. (A-B) NRK-52E cells were treated with acrolein (0-30 μM) for 1 h. Intracellular and mitochondrial ROS production was assessed using DCF and MitoSOX Red staining assays, respectively. (C-D) Cells were treated with acrolein (0-30 μM) or carbonyl cyanide m-chlorophenyl hydrazone (CCCP; 100 μM) for 1 hour. The mitochondrial membrane potential (MMP) and ATP content were measured using the JC-1 assay for MMP and ATP bioluminescence assay kits, respectively. (E-F) Oxygen consumption rate (OCR) was analyzed using the Seahorse XFe24 Metabolic Flux Analyzer. Group data from original tracings illustrate OCR changes in response to sequential administration of oligomycin (1 μM), carbonyl cyanide 4-(trifluoromethoxy)phenylhydrazone (FCCP; 2 μM), and rotenone/antimycin A (1 μM). (F) Bar graphs represent data from 3 independent experiments. (G) ATP production rate from mitochondria and glycolysis was simultaneously quantified using Seahorse ATP Real-Time rate assay. (H-J) Primary renal tubule cells were isolated from Aldh2 wild-type mice, then subjected to treatment with acrolein (0-30 μM) for 1 hour. OCR was analyzed using the Seahorse XFe24 Metabolic Flux Analyzer. Group data from original tracings illustrate OCR changes in response to sequential administration of

oligomycin (1 μM), FCCP (1 μM), and rotenone/antimycin A (1 μM). (I) Bar graphs represent data from 3 independent experiments. (J) ATP production rate from glycolysis and mitochondria was simultaneously quantified using Seahorse ATP Real-Time rate assay. Data are presented as mean ± SEM. Statistical significance was determined using Kruskal-Wallis tests, and two-tailed p-values are indicated. *$p < 0.05$; **$p<0.01$, ***$p<0.005$ compared to vehicle treatment. Abbreviations: ALDH2: aldehyde dehydrogenase 2; ATP: adenosine triphosphate; CCCP: carbonyl cyanide m-chlorophenyl hydrazone; FCCP: carbonyl cyanide 4-(trifluoromethoxy)phenylhydrazone; MMP; mitochondrial membrane potential; OCR: oxygen consumption rate.

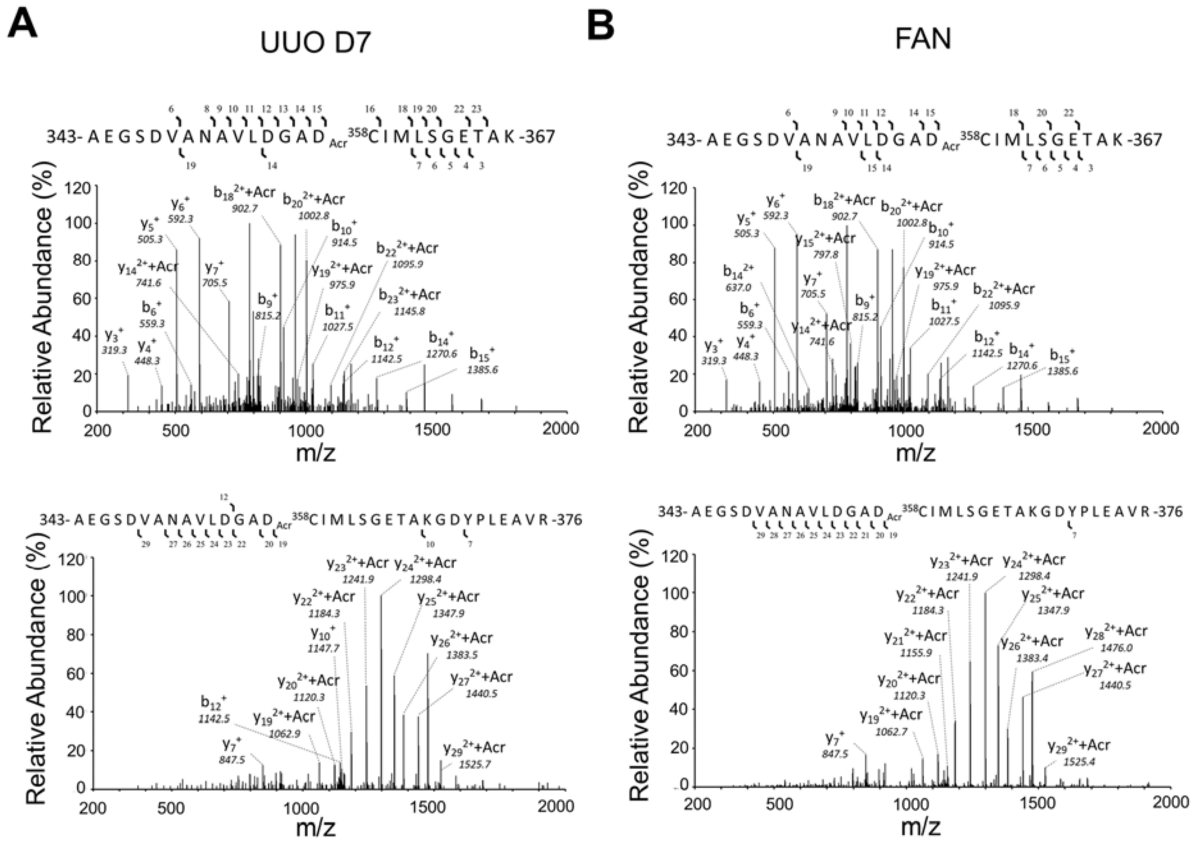

Supplementary Figure 3. Analysis of PKM2 modification and activity in murine kidneys through UUO or FAN. (A-B) Tandem mass spectrum depicting acrolein-modified PKM2 peptide in (A) UUO day 7 mice or (B) FA injection day 28 mice. The position of the peptide in the protein is indicated by its sequence's N- and C-termini numbers. The identified b- and y-ion series are marked above and below the peptide sequence. Acrolein modification is inferred at Cys358 and indicated as "Acr" on the front (Cys358). Abbreviations: FAN: folic acid nephropathy; PKM2: pyruvate kinase M2; UUO: unilateral ureteral obstruction.

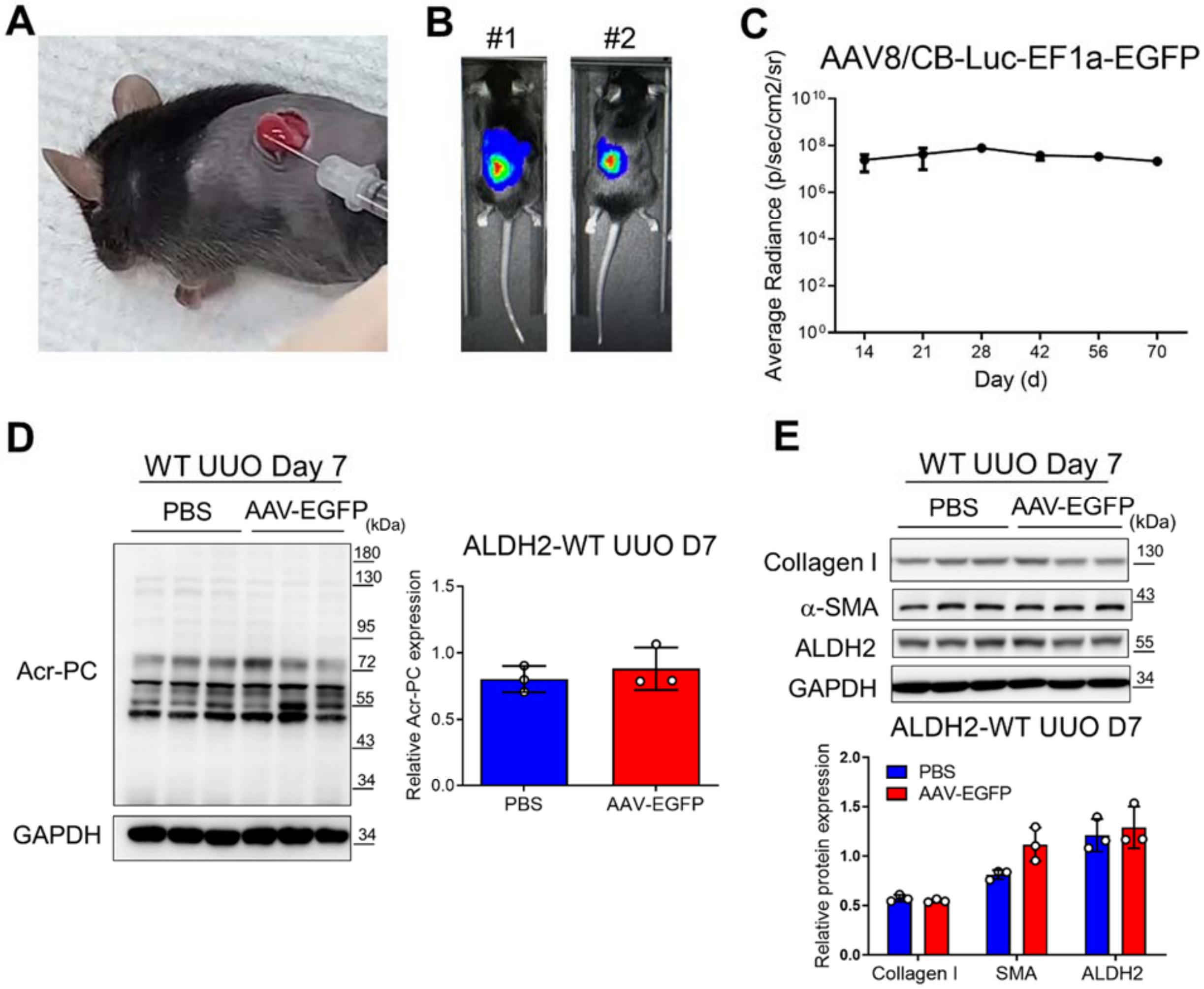

Supplementary Figure 4. The efficiency of kidney transduction using adeno-associated virus vector (AAV)-directed reporter gene via subcapsular (SC) injection. (A) Illustration of the SC injection method for delivering AAV to transduce cells in the kidney. Mice received SC injections of $2x10^{11}$ GC of AAV8/CB-Luc-EF1a-EGFP. (B) Kidney transduction efficacy was assessed through *in vivo* luminescence measurements on day 28. (C) Luminescent signals were recorded from days 10 to 70 (n = 3). Luminescence is quantified as average radiance, p/sec/cm^2/sr. (D-E) ALDH2*1 (wild-type) mice (n=3) were subjected to SC injections of PBS or $2x10^{11}$ GC of AAV8-EGFP for 28 days, followed by UUO surgery for 7 days. (D) Western blot analysis of Acr-PC in kidney tissues of mice is illustrated in the left panel, with quantification of these proteins in the right panel. (E) Western blot analysis of collagen 1, α-smooth muscle actin (α-SMA), and ALDH2 in kidney tissues of mice is illustrated in the upper panel, with quantification of these proteins in the lower panel. Data are presented as mean ± SD. Statistical significance was determined using Mann-Whitney U tests, and two-tailed p-values are indicated. Abbreviations: Acr-PC: acrolein-protein conjugates; ALDH2: aldehyde dehydrogenase 2; α-SMA: α-smooth muscle actin; UUO: unilateral ureteral obstruction.

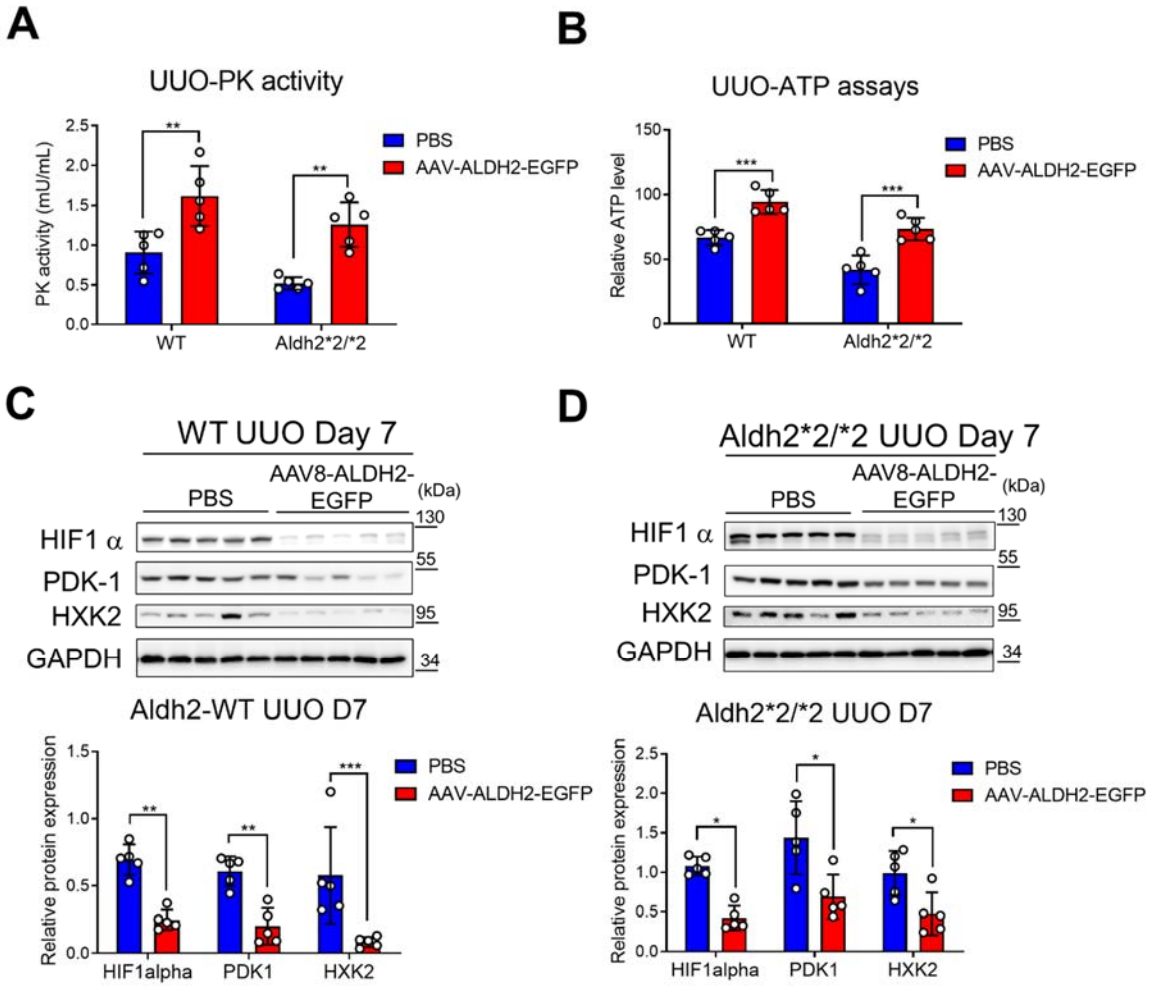

Supplementary Figure 5. Impact of adeno-associated virus vector (AAV)-directed ALDH2 gene on pyruvate kinase (PK) activity, pyruvate kinase M2 (PKM2) downstream signaling, and mitochondrial ATP content after unilateral ureteral obstruction (UUO). Aldh2*1 (wild type, WT) mice and Aldh2*2/*2 mice were subjected to subcapsular (SC) injections of $2x10^{11}$ GC of AAV8-ALDH2-EGFP for 28 days, followed by UUO surgery for 7 days. (A) PK activity in the kidney tissues of mice was determined using a Pyruvate Kinase Assay Kit (ab83432). (B) ATP content of mitochondria isolated from kidney tissues of mice was measured by ATP bioluminescence assay kit. (C-D) The upper panel displays Western blot analysis of HIF-1α, HXK2, and PDK-1 in kidney tissues of wild-type (the left panel) and Aldh2*2/*2 (the right panel) mice, with quantification of these proteins shown in the lower panel. Data is presented as mean ± SD. Statistical significance was determined using Kruskal-Wallis tests, with two-tailed p values indicated. *p<0.05, **p<0.01, ***p<0.001 compared with vehicle treatment. Abbreviations: Aldh2: aldehyde dehydrogenase 2; HXK2: hexokinase 2; HIF-1α: hypoxia-inducible factor 1 alpha; PDK1: pyruvate dehydrogenase kinase 1; UUO: unilateral ureteral obstruction.

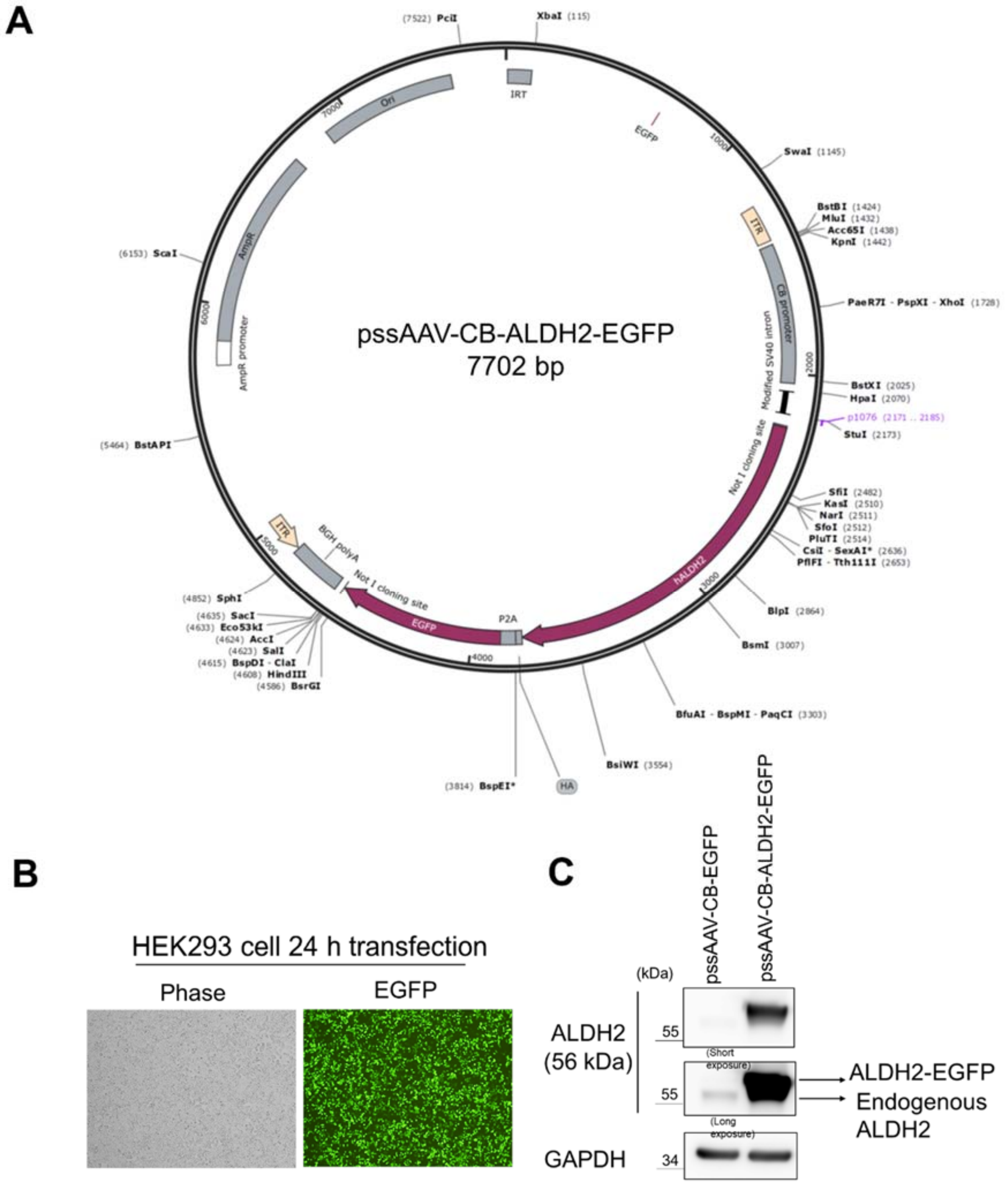

Supplementary Figure 6. Construction and expression of ALDH2-EGFP in HEK293 cells. (A) The human *ALDH2* gene was PCR amplified from HEK293 cell cDNA with the wild-type *ALDH2* sequence and cloned into pJET1.2/blunt using the CloneJET PCR Cloning kit (Thermo Scientific). The *ALDH2* PCR fragment and HA-P2A synthetic oligonucleotides were assembled into the AAV8/CB-EGFP plasmid using the In-Fusion® HD Cloning Kit (Takara Bio USA, Inc.). The sequence of pssAAV-CB-ALDH2-EGFP was confirmed through Sanger sequencing, with the map displaying restriction enzyme sites. (B-C) HEK293 cells were transiently transfected with AAV8-CB-ALDH2-EGFP, and ALDH2 expression was verified through (B) immunofluorescent staining and (C) Western blot analysis after 24 h. Abbreviations: ALDH2: aldehyde dehydrogenase 2

References

1. Tervaert TW, Mooyaart AL, Amann K, Cohen AH, Cook HT, Drachenberg CB, et al. Pathologic classification of diabetic nephropathy. *J Am Soc Nephrol.* 2010;21(4):556-63.
2. Hoshino J, Mise K, Ueno T, Imafuku A, Kawada M, Sumida K, et al. A pathological scoring system to predict renal outcome in diabetic nephropathy. *Am J Nephrol.* 2015;41(4-5):337-44.
3. Huang CH, Chen YT, Lin JH, and Wang HT. Acrolein induces ribotoxic stress in human cancer cells regardless of p53 status. *Toxicol In Vitro.* 2018;52:265-71.
4. Rubin JD, Nguyen TV, Allen KL, Ayasoufi K, and Barry MA. Comparison of Gene Delivery to the Kidney by Adenovirus, Adeno-Associated Virus, and Lentiviral Vectors After Intravenous and Direct Kidney Injections. *Hum Gene Ther.* 2019;30(12):1559-71.
5. Acin-Perez R, Montales KP, Nguyen KB, Brownstein AJ, Stiles L, and Divakaruni AS. Isolation of Mitochondria from Mouse Tissues for Functional Analysis. *Methods in molecular biology.* 2023;2675:77-96.
6. Breggia AC, and Himmelfarb J. Primary mouse renal tubular epithelial cells have variable injury tolerance to ischemic and chemical mediators of oxidative stress. *Oxid Med Cell Longev.* 2008;1(1):33-8.
7. Sivandzade F, Bhalerao A, and Cucullo L. Analysis of the Mitochondrial Membrane Potential Using the Cationic JC-1 Dye as a Sensitive Fluorescent Probe. *Bio Protoc.* 2019;9(1).
8. Jhuo JY, Tong ZJ, Ku PH, Cheng HW, and Wang HT. Acrolein induces mitochondrial dysfunction and insulin resistance in muscle and adipose tissues in vitro and in vivo. *Environ Pollut.* 2023;336:122380.
9. Tong ZJ, Kuo CW, Yen PC, Lin CC, Tsai MT, Lu SH, et al. Acrolein plays a culprit role in the pathogenesis of diabetic nephropathy in vitro and in vivo. *Eur J Endocrinol.* 2022;187(4):579-92.
10. Kim JW, Tchernyshyov I, Semenza GL, and Dang CV. HIF-1-mediated expression of pyruvate dehydrogenase kinase: a metabolic switch required for cellular adaptation to hypoxia. *Cell Metab.* 2006;3(3):177-85.
11. Luo W, Hu H, Chang R, Zhong J, Knabel M, O'Meally R, et al. Pyruvate kinase M2 is a PHD3-stimulated coactivator for hypoxia-inducible factor 1. *Cell.* 2011;145(5):732-44.
12. Kuo CW, Chen DH, Tsai MT, Lin CC, Cheng HW, Tsay YG, et al. Pyruvate kinase M2 modification by a lipid peroxidation byproduct acrolein contributes to kidney fibrosis. *Front Med (Lausanne).* 2023;10:1151359.
