# Supplementary material for: Aldehyde dehydrogenase 2 preserves kidney function by countering acrolein-induced metabolic and mitochondrial dysfunction
Source: JCI Insight. 2024 Oct 8;9(19):e179871. doi: 10.1172/jci.insight.179871 (PMC11466184; doi:10.1172/jci.insight.179871)

Figure 2C

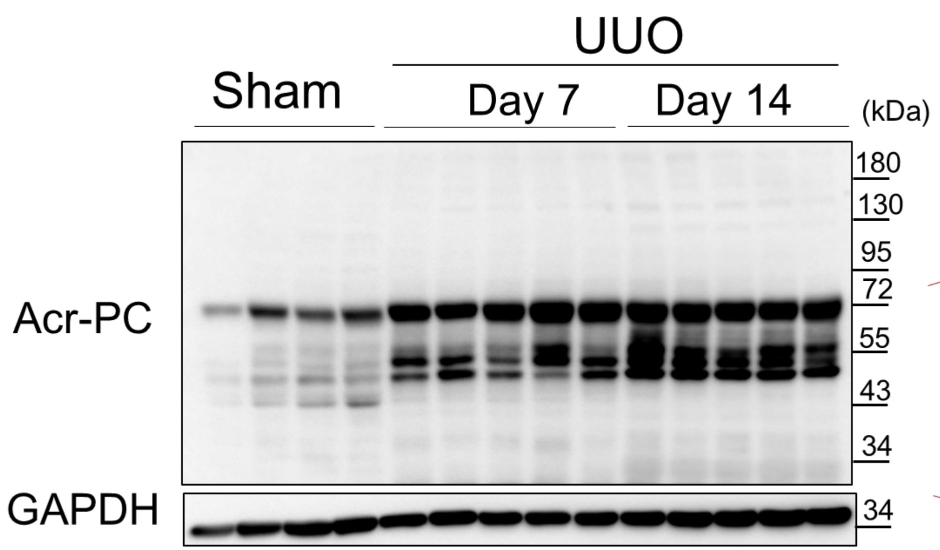

UUO
Sham
Day 7
Day 14
(kDa)
Acr-PC
GAPDH
180
130
95
72
55
43
34
34

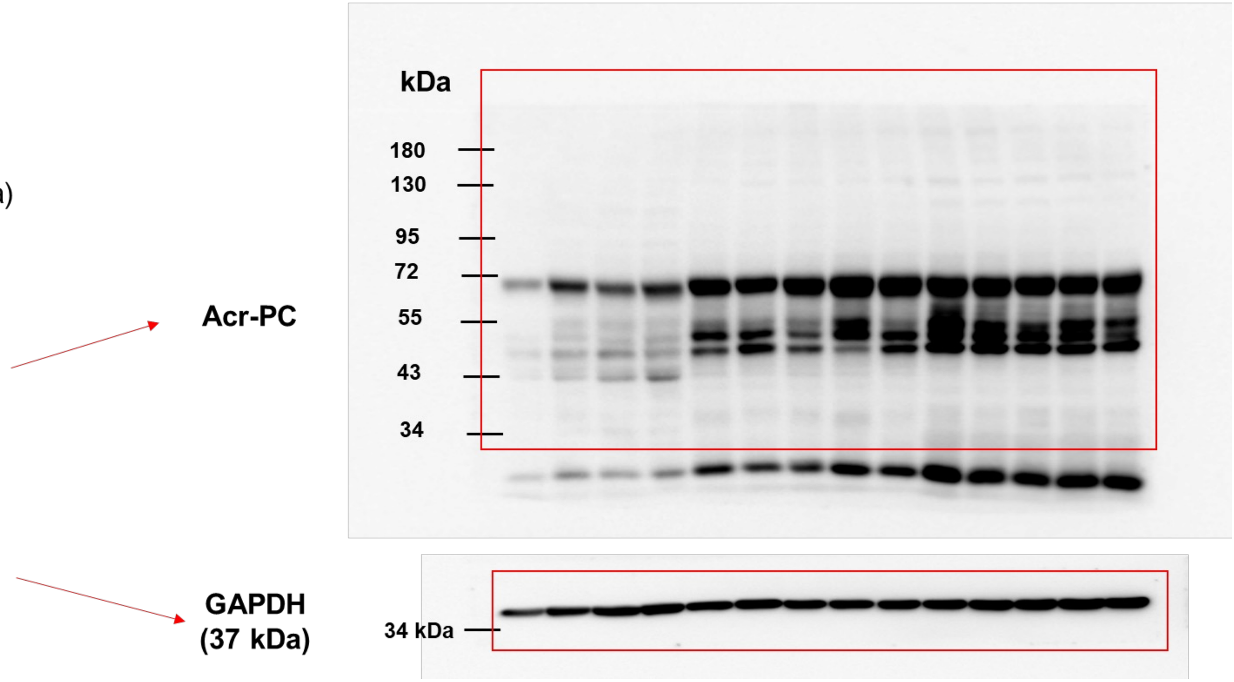

Acr-PC
GAPDH
(37 kDa)
kDa
180
130
95
72
55
43
34
34 kDa

Figure 2D

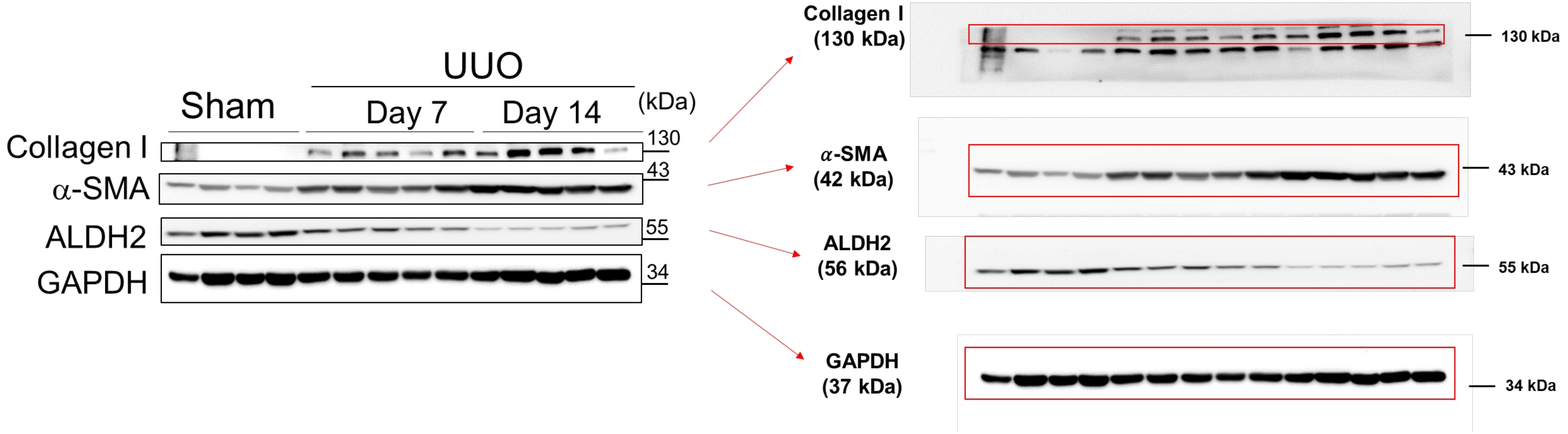

UUO
Sham
Day 7
Day 14
(kDa)
Collagen I
α-SMA
ALDH2
GAPDH
130
43
55
34
Collagen I
(130 kDa)
α-SMA
(42 kDa)
ALDH2
(56 kDa)
GAPDH
(37 kDa)
130 kDa
43 kDa
55 kDa
34 kDa

Figure 3D

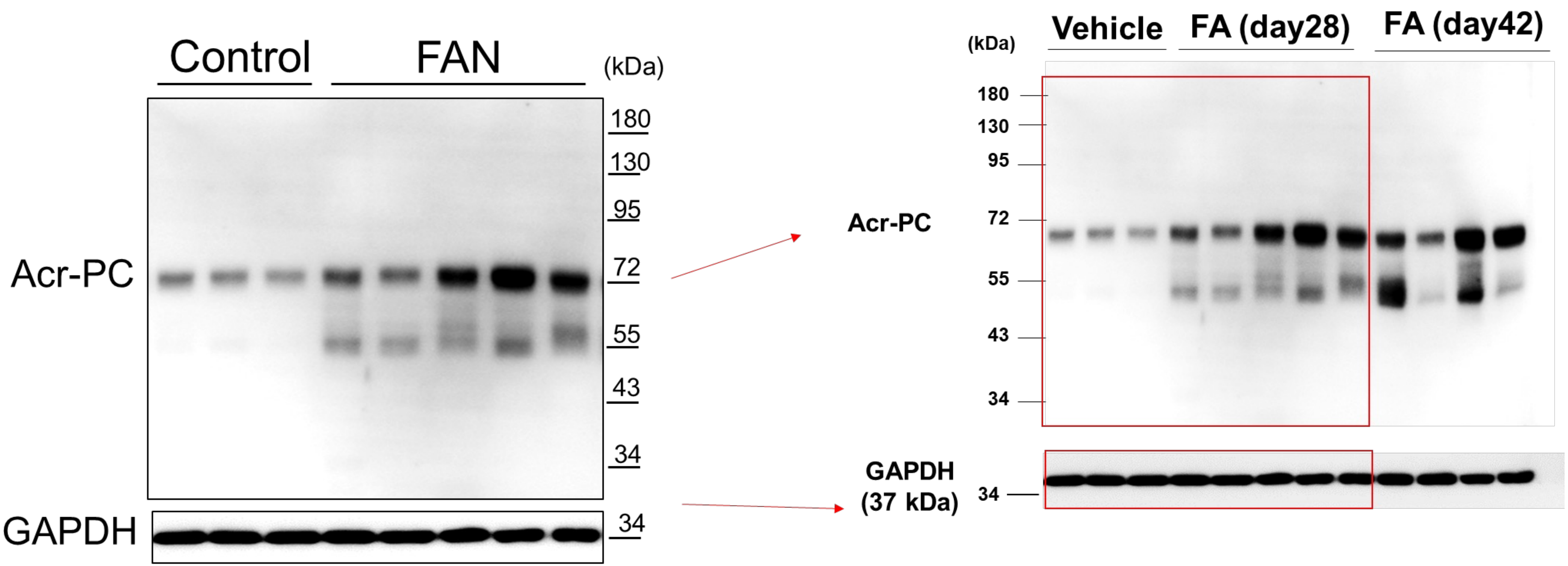

Control
FAN
(kDa)
180
130
95
72
55
43
34
Acr-PC
GAPDH
34
Vehicle
FA (day28)
FA (day42)
(kDa)
180
130
95
72
55
43
34
Acr-PC
GAPDH
(37 kDa)
34

Figure 3E

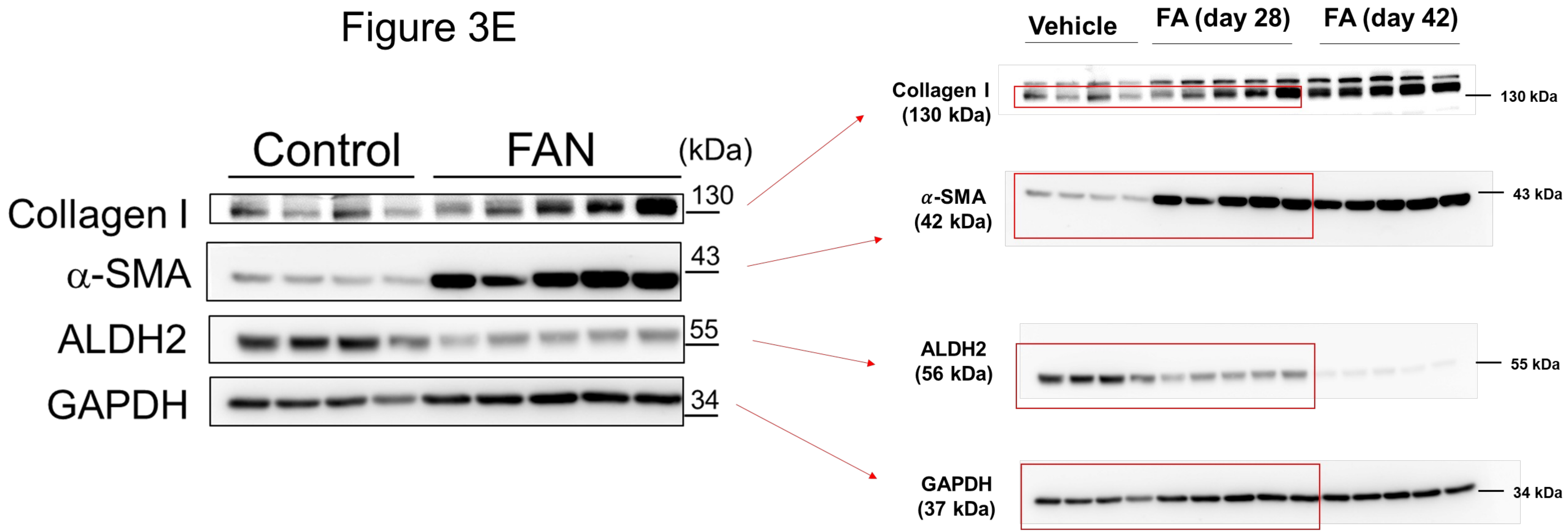
Control
FAN
(kDa)
Collagen I
α-SMA
ALDH2
GAPDH
130
43
55
34
Vehicle
FA (day 28)
FA (day 42)
Collagen I
(130 kDa)
α-SMA
(42 kDa)
ALDH2
(56 kDa)
GAPDH
(37 kDa)
130 kDa
43 kDa
55 kDa
34 kDa

Figure 4B

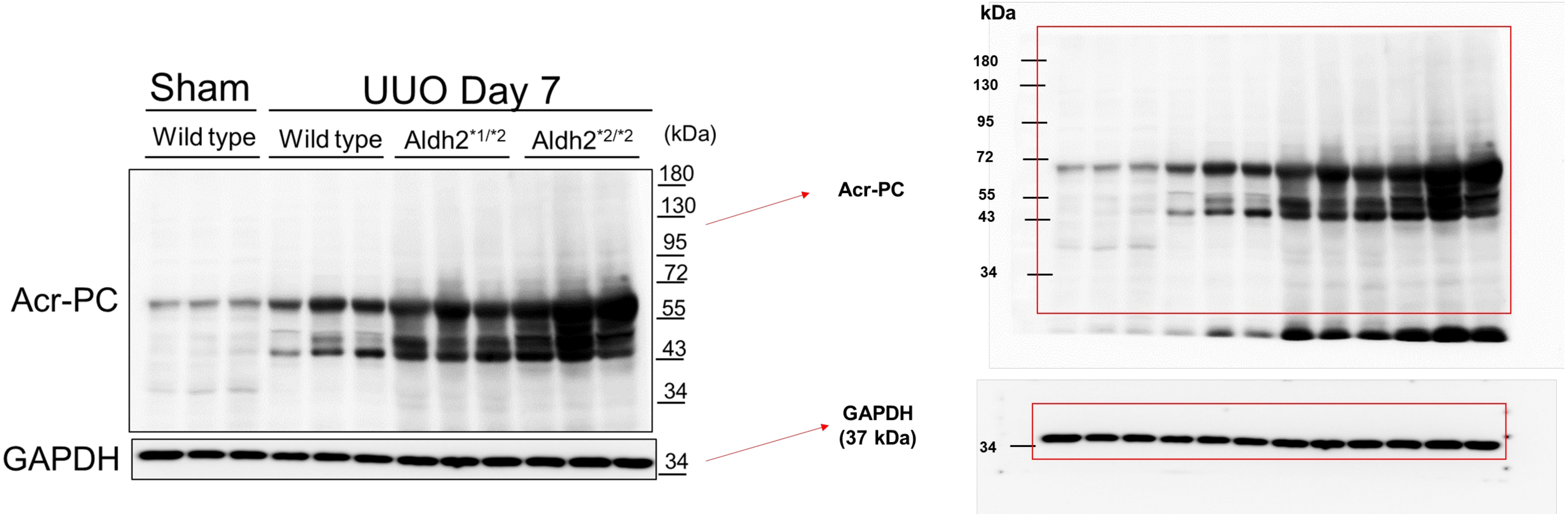
Sham
UUO Day 7
Wild type
Wild type
Aldh2*1/*2
Aldh2*2/*2
(kDa)
Acr-PC
180
130
95
72
55
43
34
GAPDH
34
Acr-PC
GAPDH
(37 kDa)
kDa
180
130
95
72
55
43
34
34

Figure 4C

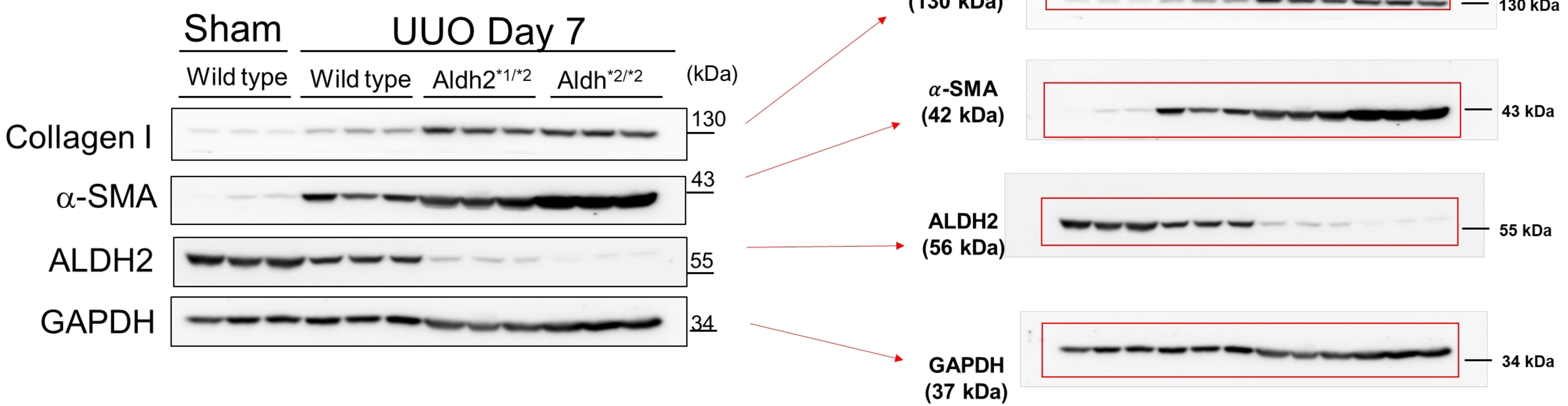
Sham
UUO Day 7
Wild type
Wild type
Aldh2*1/*2
Aldh*2/*2
(kDa)
Collagen I
α-SMA
ALDH2
GAPDH
130
43
55
34
Collagen I (130 kDa)
α-SMA (42 kDa)
ALDH2 (56 kDa)
GAPDH (37 kDa)
130 kDa
43 kDa
55 kDa
34 kDa

Figure 5C

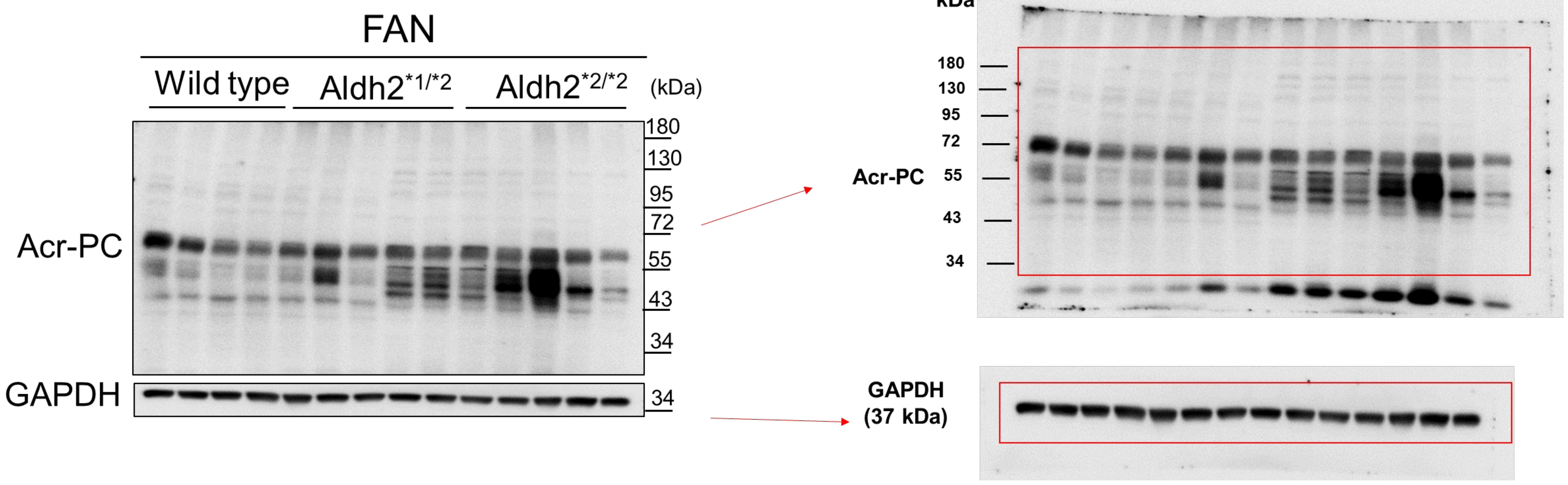

FAN
Wild type
Aldh2*1/*2
Aldh2*2/*2
(kDa)
180
130
95
72
55
43
34
Acr-PC
GAPDH
34
kDa
180
130
95
72
55
43
34
Acr-PC
GAPDH
(37 kDa)

Figure 5D

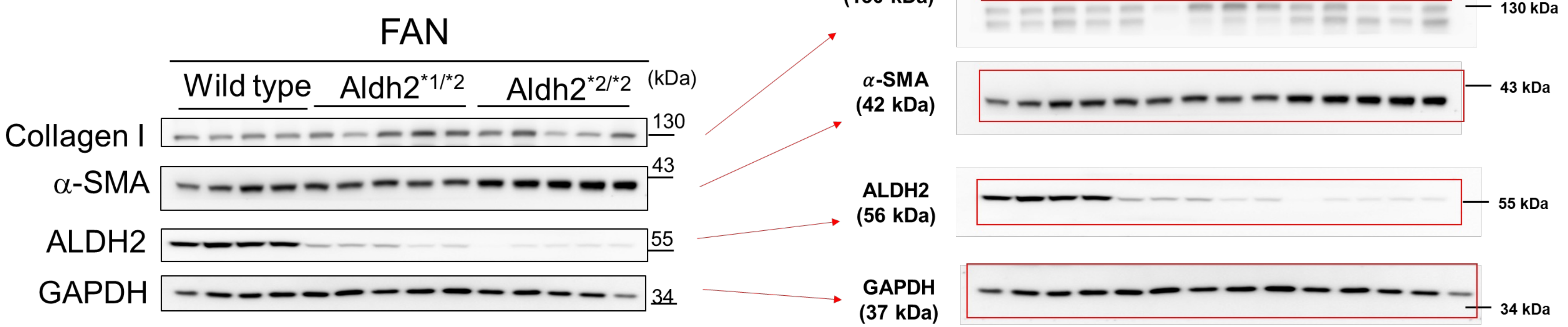
FAN
Wild type
Aldh2*1/*2
Aldh2*2/*2
(kDa)
Collagen I
α-SMA
ALDH2
GAPDH
130
43
55
34
kDa
Collagen I
(130 kDa)
α-SMA
(42 kDa)
ALDH2
(56 kDa)
GAPDH
(37 kDa)
130 kDa
43 kDa
55 kDa
34 kDa

Figure 6A

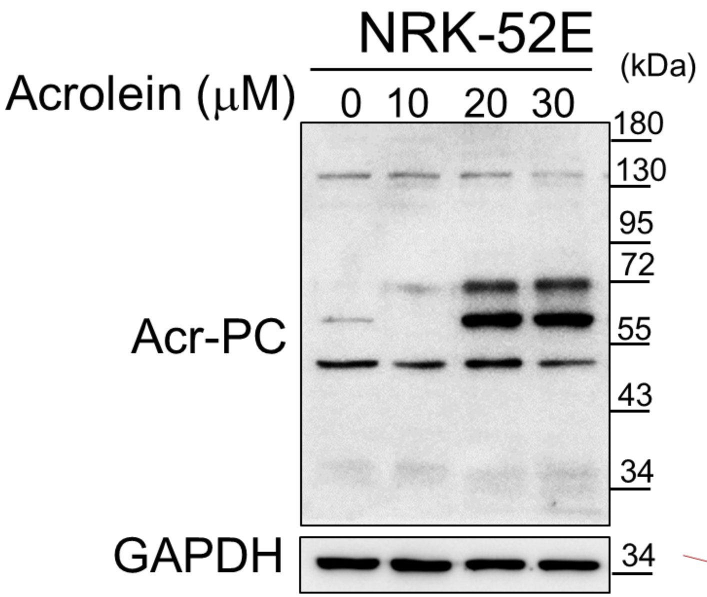
NRK-52E
Acrolein (μM)
0 10 20 30
(kDa)
180
130
95
72
55
43
34
Acr-PC
GAPDH
34

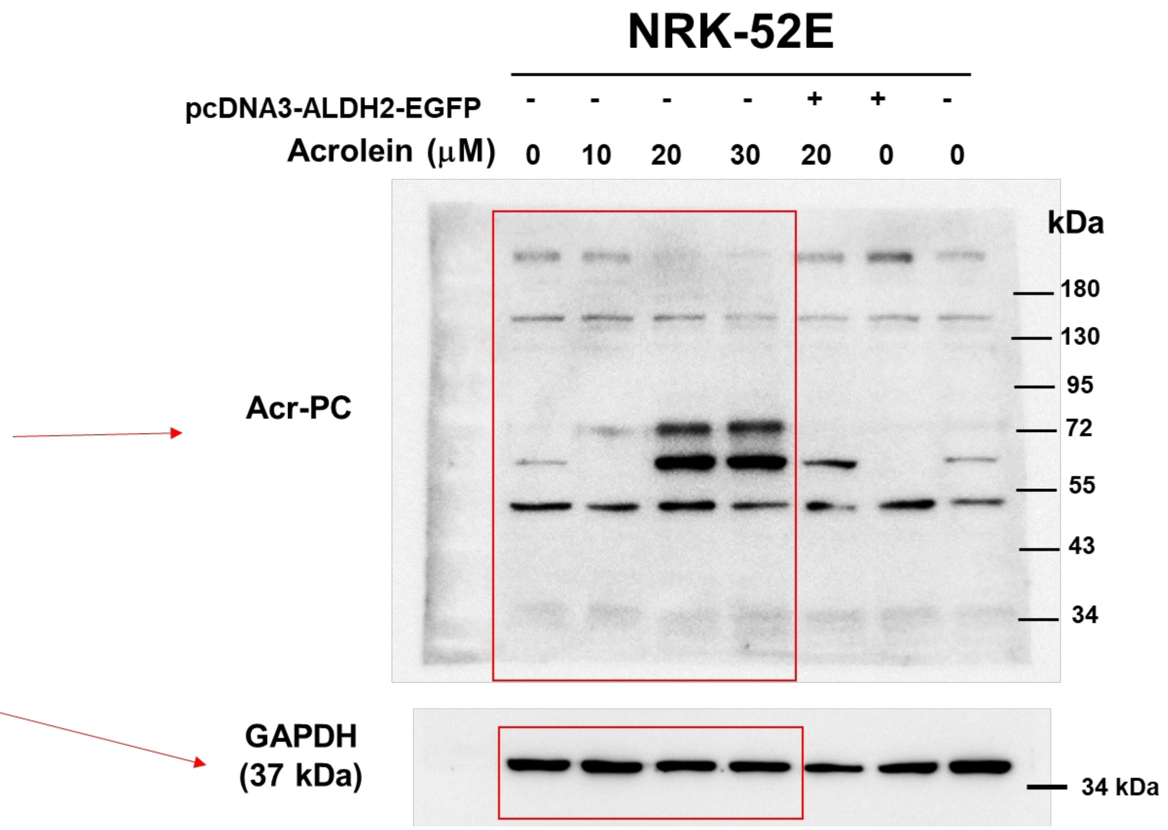
NRK-52E
pcDNA3-ALDH2-EGFP
- - - - + + -
Acrolein (μM)
0 10 20 30 20 0 0
kDa
180
130
95
72
55
43
34
Acr-PC
GAPDH
(37 kDa)
34 kDa

Figure 6E

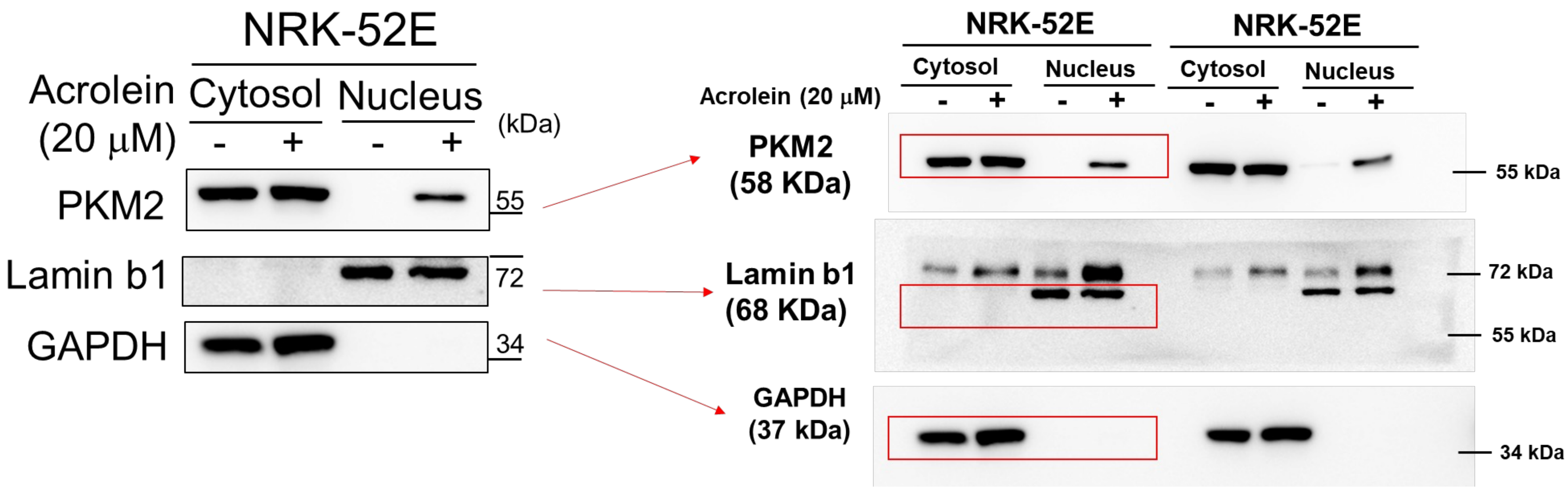

NRK-52E
Acrolein (20 μM)
Cytosol
Nucleus
- + - +
(kDa)
PKM2
Lamin b1
GAPDH
55
72
34
NRK-52E
NRK-52E
Cytosol
Nucleus
Cytosol
Nucleus
Acrolein (20 μM)
- + - + - + - +
PKM2 (58 KDa)
Lamin b1 (68 KDa)
GAPDH (37 kDa)
55 kDa
72 kDa
55 kDa
34 kDa

Figure 6F

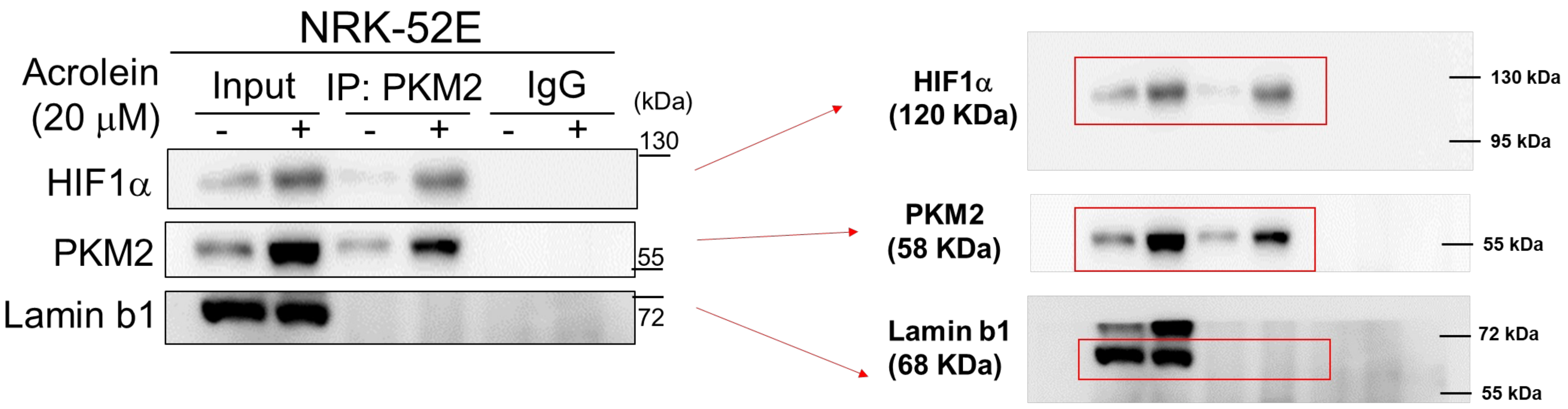
NRK-52E
Acrolein (20 μM)
Input
IP: PKM2
IgG
- + - + - +
(kDa)
130
55
72
HIF1α
PKM2
Lamin b1
HIF1α (120 KDa)
PKM2 (58 KDa)
Lamin b1 (68 KDa)
130 kDa
95 kDa
55 kDa
72 kDa
55 kDa

Figure 6H

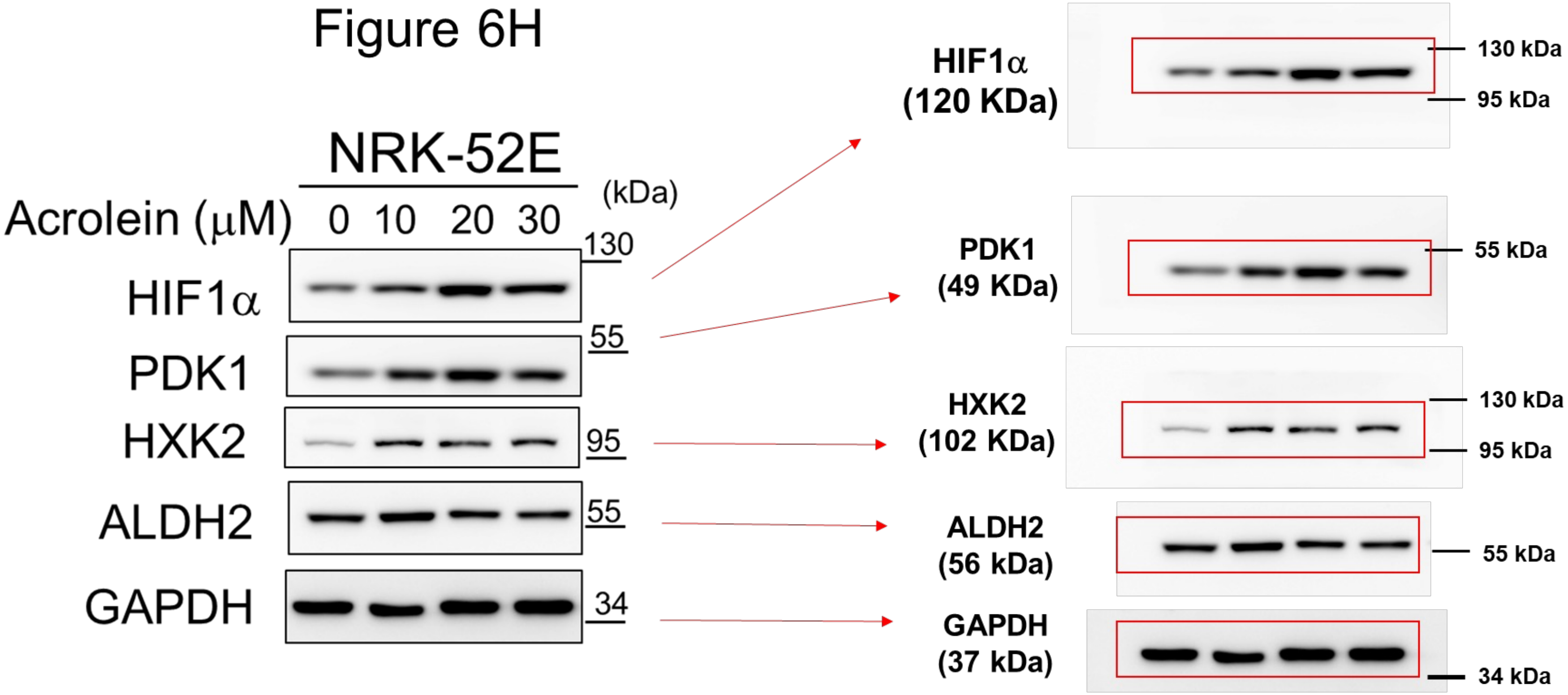
NRK-52E
Acrolein (μM) 0 10 20 30
(kDa)
HIF1α
PDK1
HXK2
ALDH2
GAPDH
130
55
95
55
34
HIF1α (120 KDa)
130 kDa
95 kDa
PDK1 (49 KDa)
55 kDa
HXK2 (102 KDa)
130 kDa
95 kDa
ALDH2 (56 kDa)
55 kDa
GAPDH (37 kDa)
34 kDa

Figure 6I

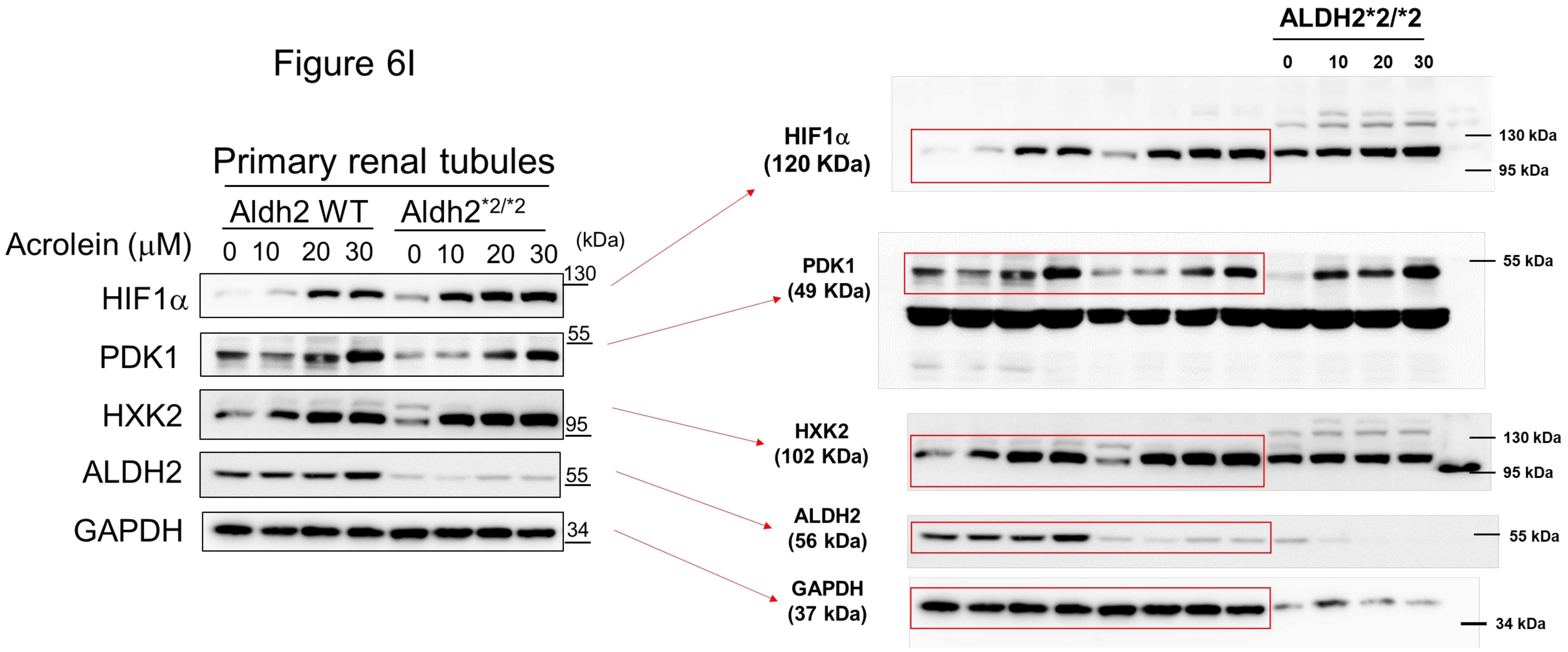

Primary renal tubules
Aldh2 WT
Aldh2*2/*2
Acrolein (μM)
0 10 20 30 0 10 20 30
(kDa)
HIF1α
PDK1
HXK2
ALDH2
GAPDH
130
55
95
55
34
ALDH2*2/*2
0 10 20 30
HIF1α (120 KDa)
130 kDa
95 kDa
PDK1 (49 KDa)
55 kDa
HXK2 (102 KDa)
130 kDa
95 kDa
ALDH2 (56 kDa)
55 kDa
GAPDH (37 kDa)
34 kDa

Figure 6K

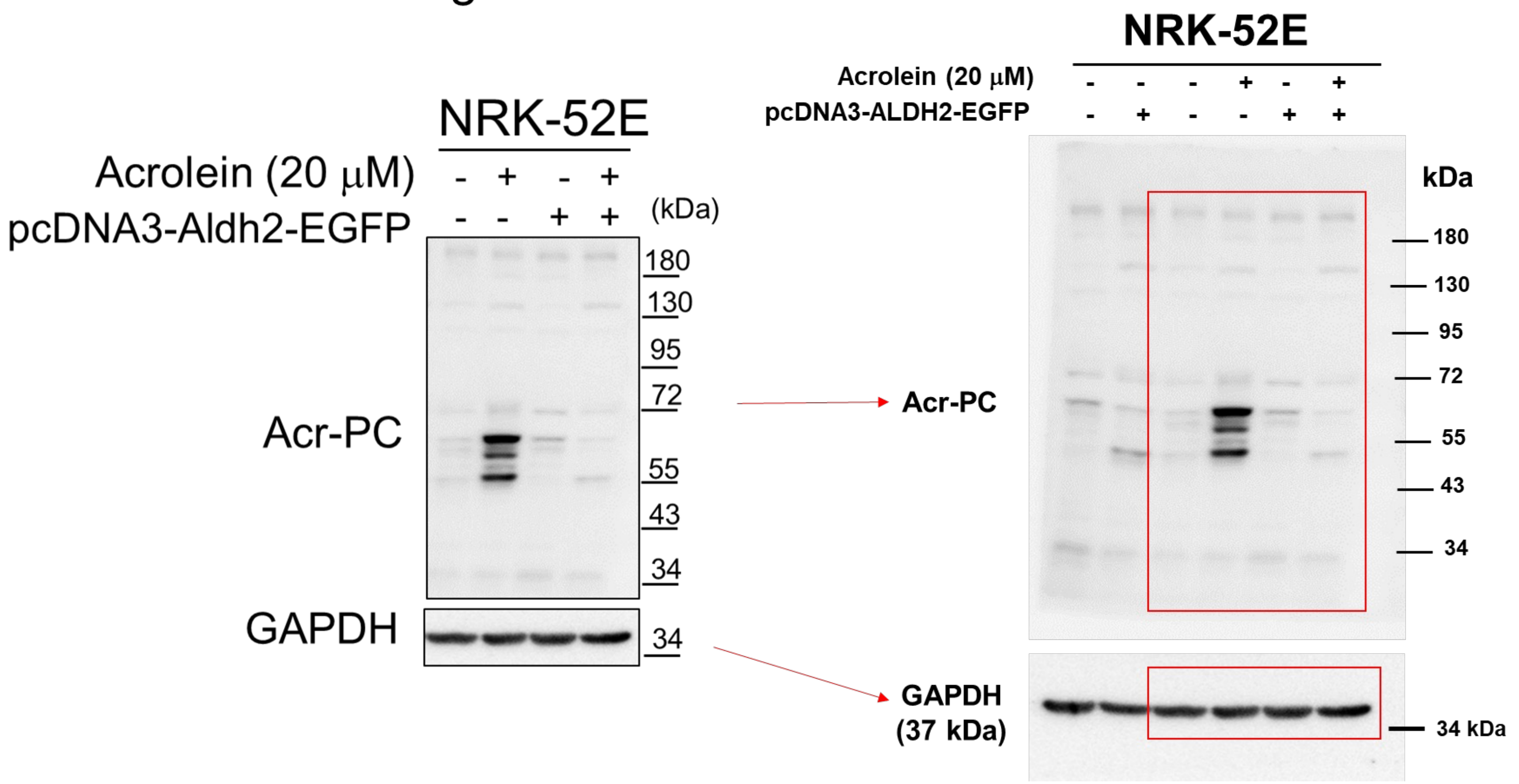

NRK-52E
Acrolein (20 µM)
pcDNA3-Aldh2-EGFP
(kDa)
180
130
95
72
55
43
34
Acr-PC
GAPDH
34
NRK-52E
Acrolein (20 µM)
pcDNA3-ALDH2-EGFP
kDa
180
130
95
72
55
43
34
Acr-PC
GAPDH
(37 kDa)
34 kDa

Figure 6L

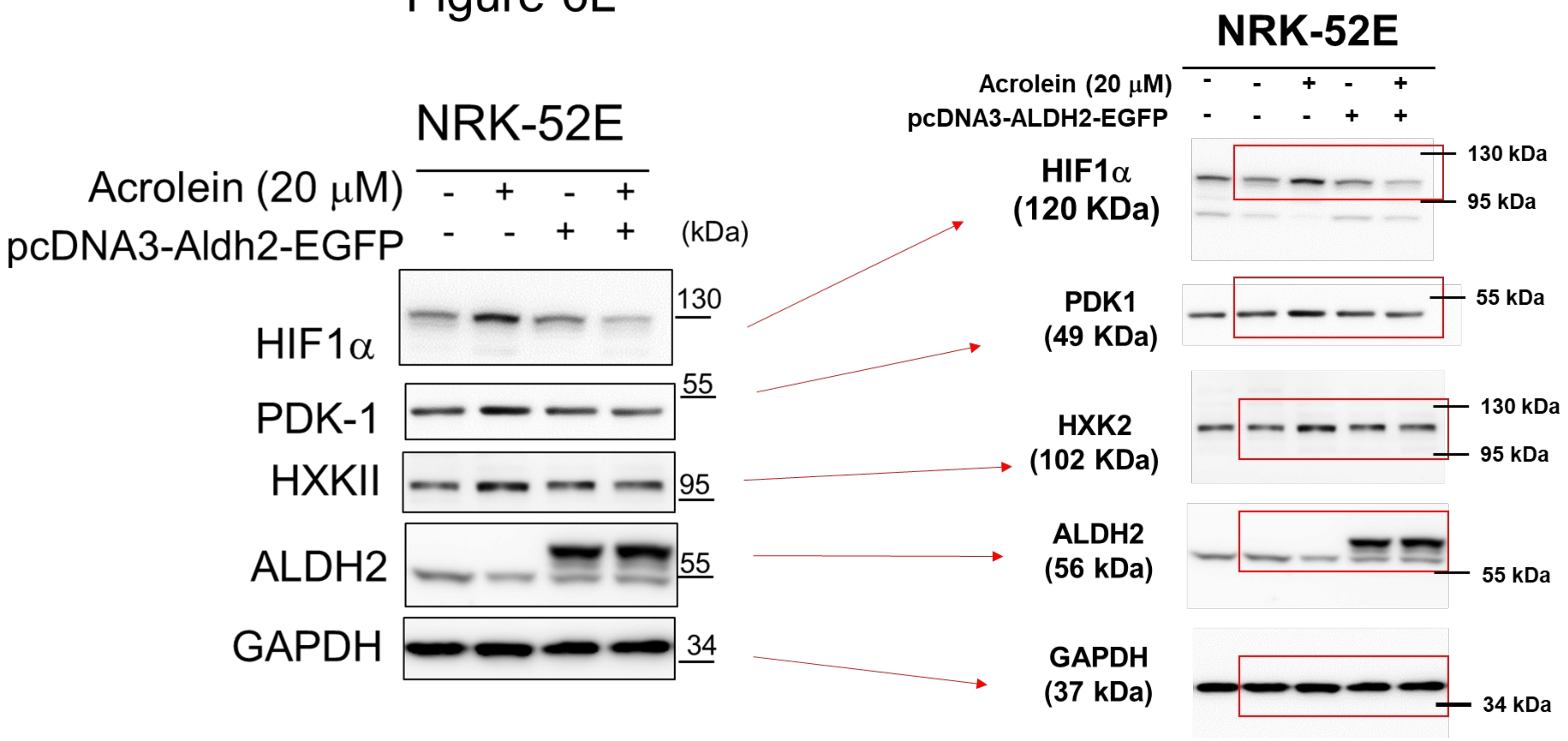

NRK-52E
Acrolein (20 μM) - + - +
pcDNA3-Aldh2-EGFP - - + +
(kDa)
HIF1α
130
PDK-1
55
HXKII
95
ALDH2
55
GAPDH
34
NRK-52E
Acrolein (20 μM) - - + - +
pcDNA3-ALDH2-EGFP - - - + +
HIF1α (120 KDa)
130 kDa
95 kDa
PDK1 (49 KDa)
55 kDa
HXK2 (102 KDa)
130 kDa
95 kDa
ALDH2 (56 kDa)
55 kDa
GAPDH (37 kDa)
34 kDa

Figure 7B

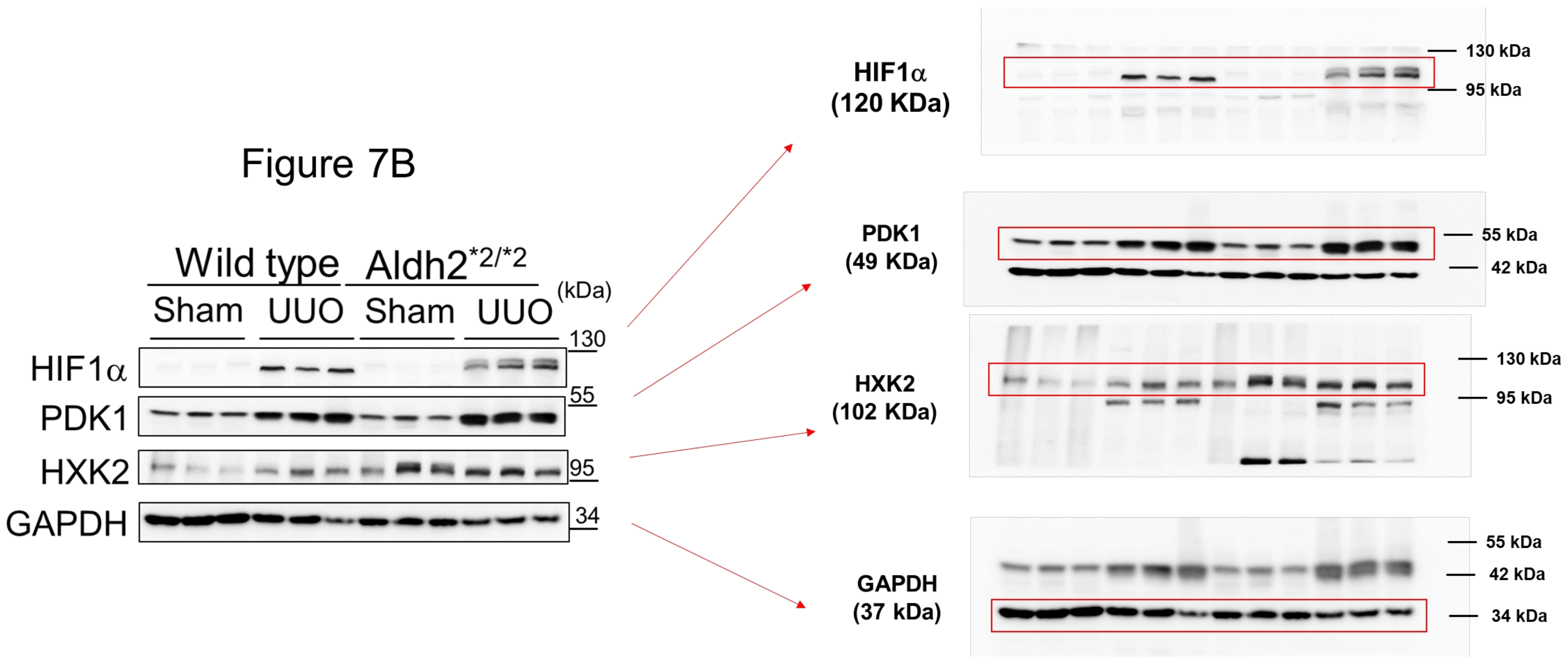

Wild type
Aldh2*2/*2
Sham
UUO
Sham
UUO
(kDa)
HIF1α
PDK1
HXK2
GAPDH
130
55
95
34
HIF1α (120 KDa)
PDK1 (49 KDa)
HXK2 (102 KDa)
GAPDH (37 kDa)
130 kDa
95 kDa
55 kDa
42 kDa
130 kDa
95 kDa
55 kDa
42 kDa
34 kDa

Figure 7D left panel

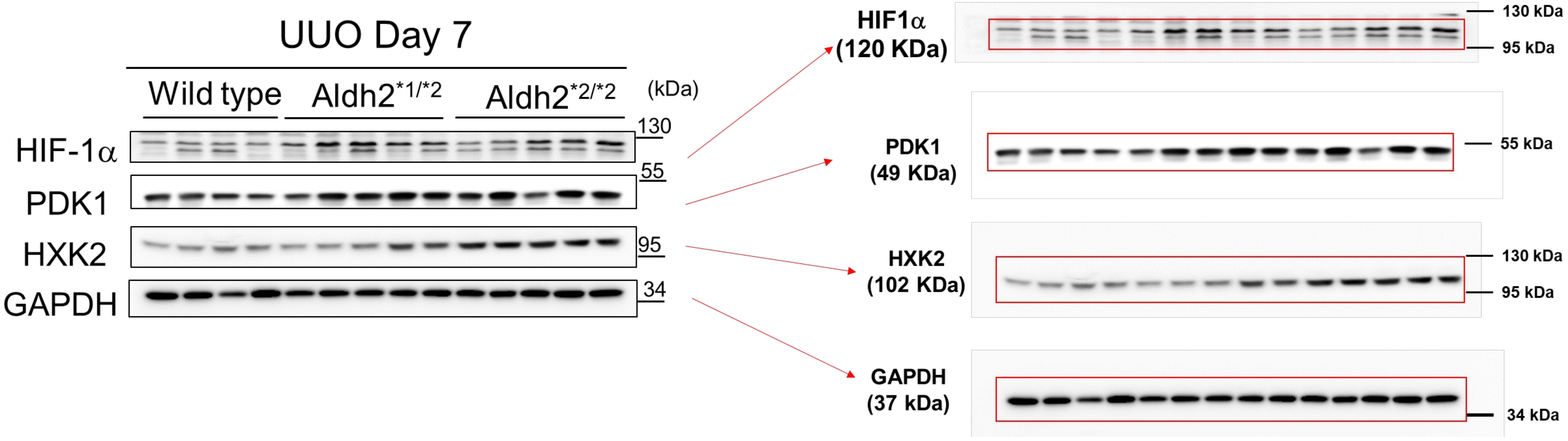

Figure 7D right panel

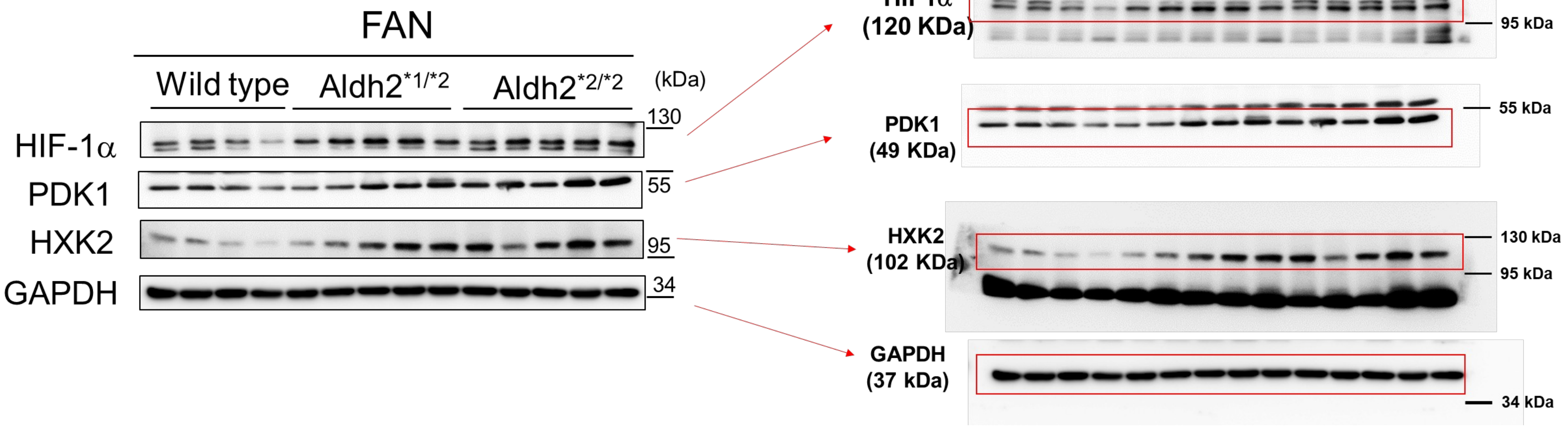

Figure 7E

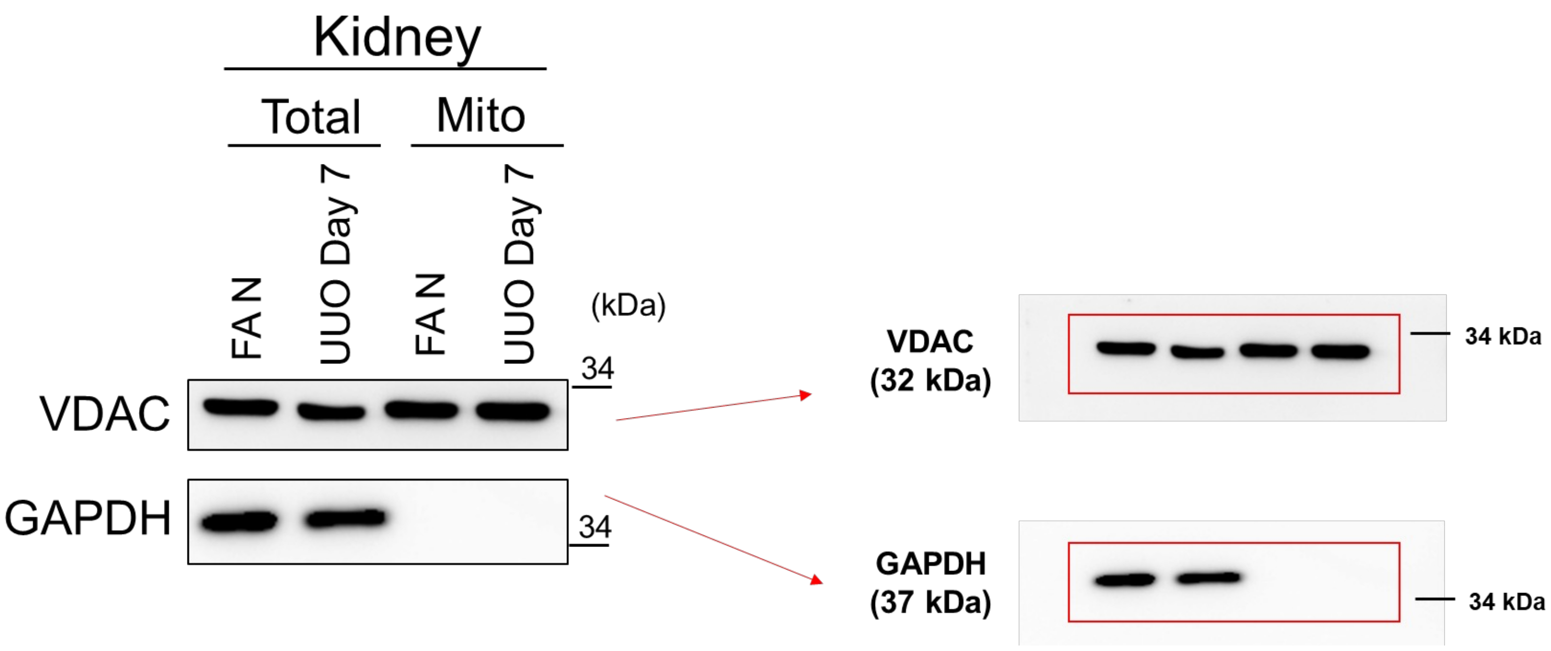
Kidney
Total
Mito
FA N
UUO Day 7
FA N
UUO Day 7
(kDa)
34
VDAC
GAPDH
34
VDAC
(32 kDa)
34 kDa
GAPDH
(37 kDa)
34 kDa

Figure 8C upper panel

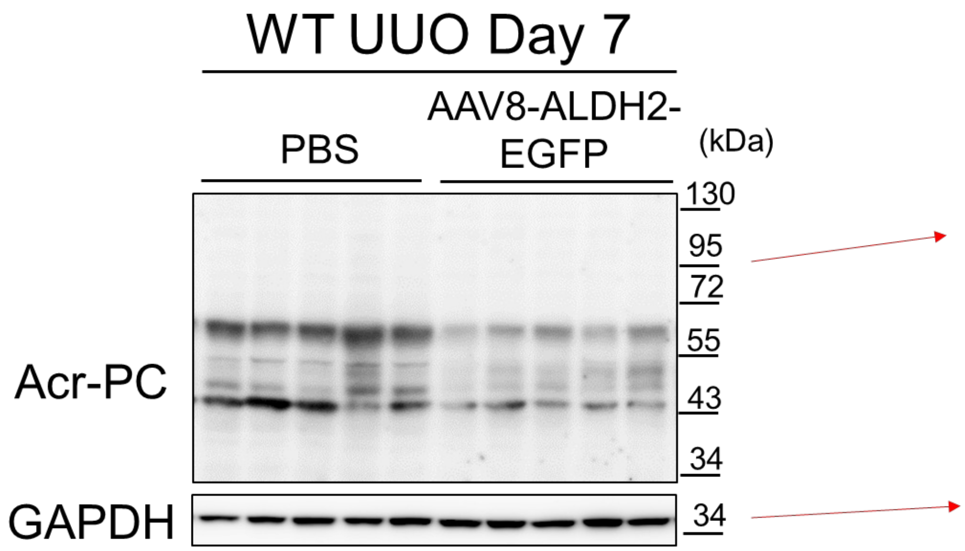

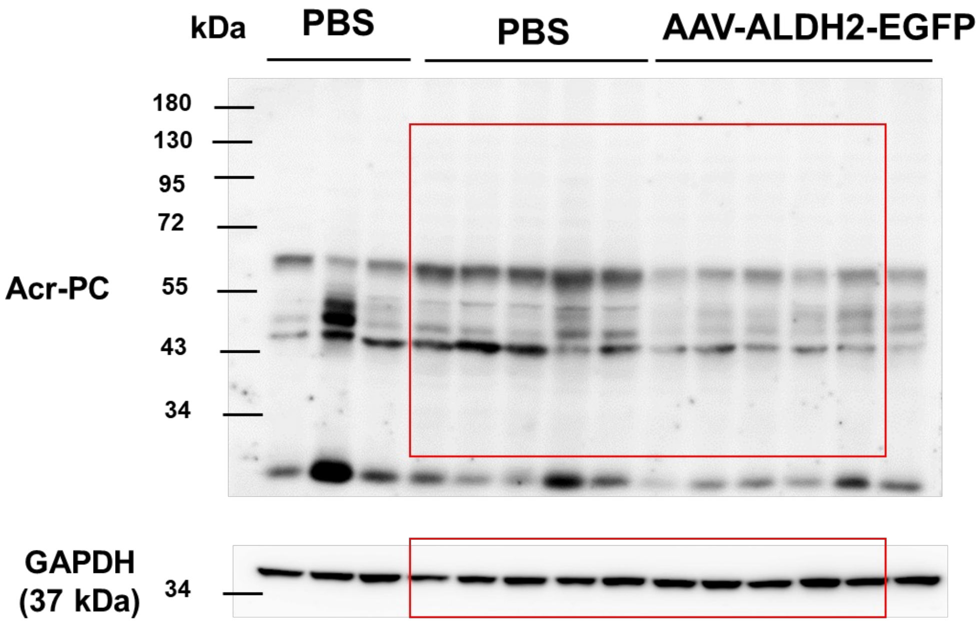

Figure 8C lower panel

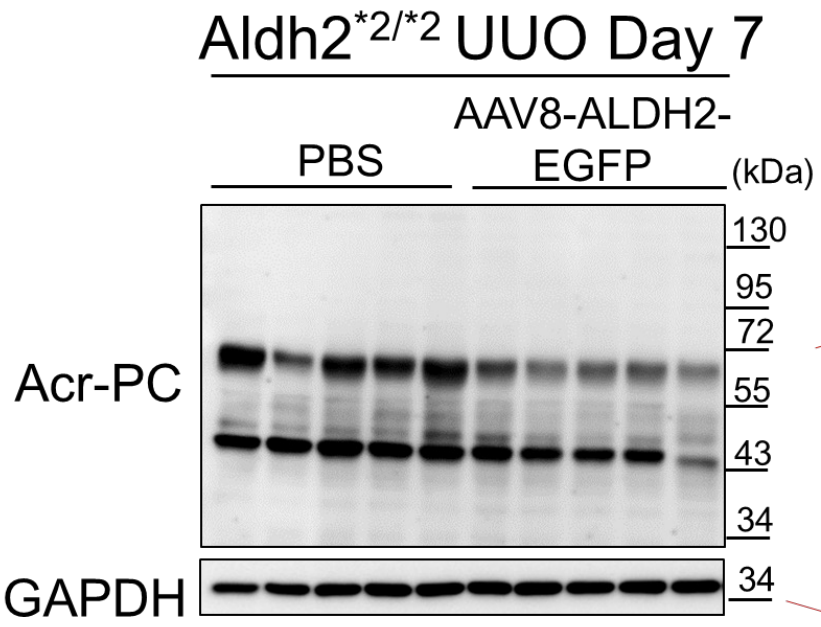

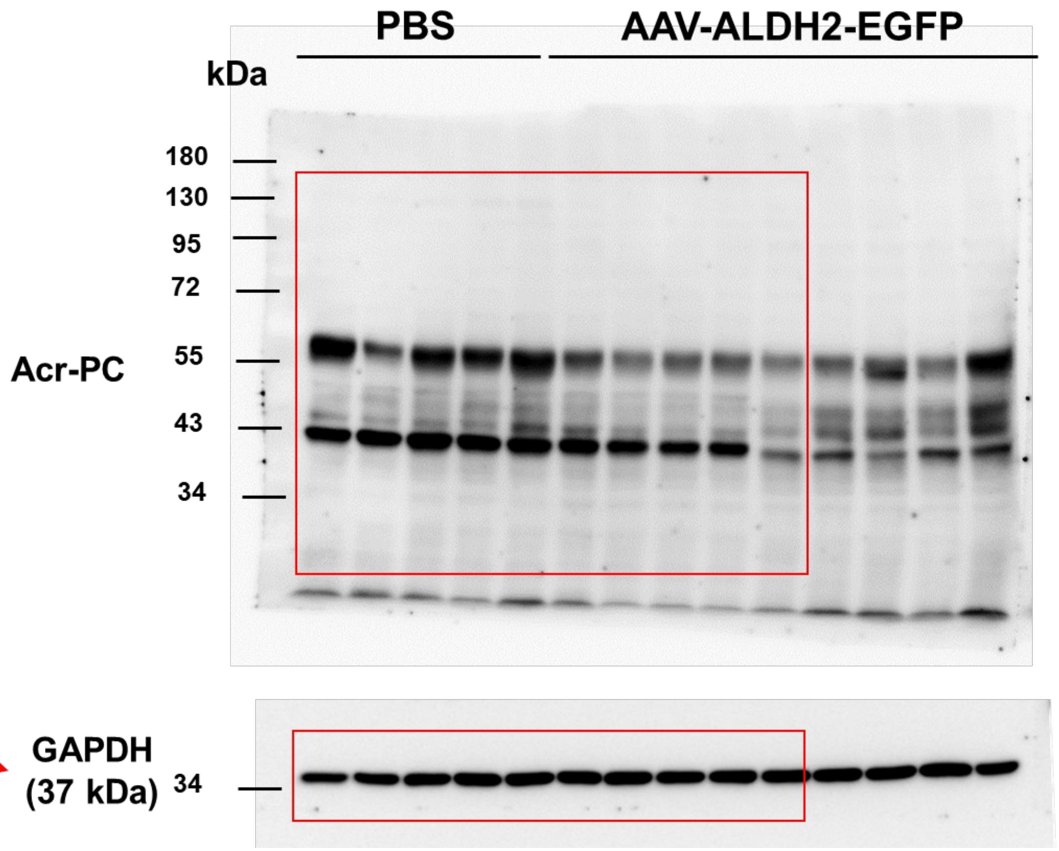

Figure 8D left panel

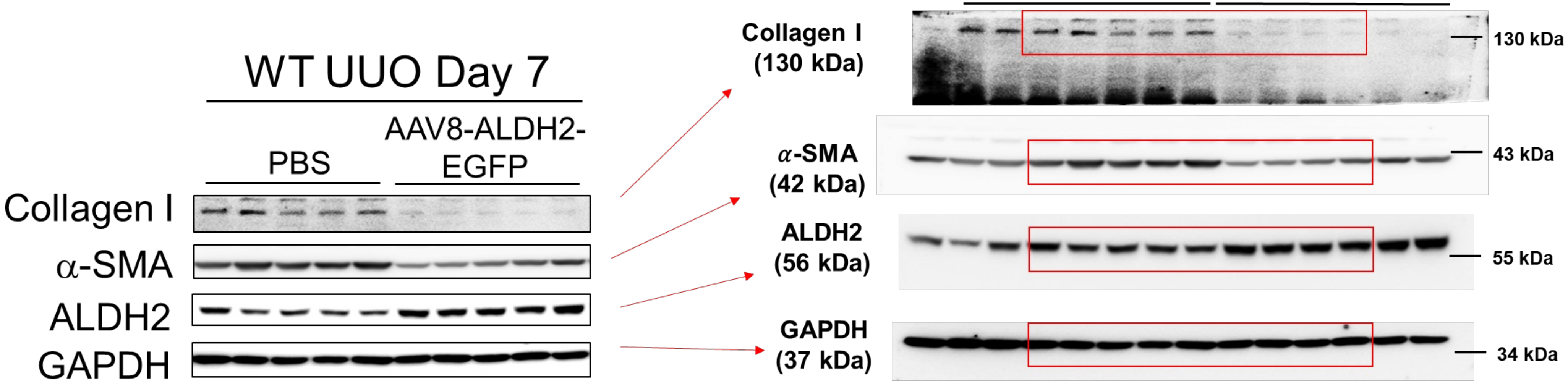

Figure 8D right panel

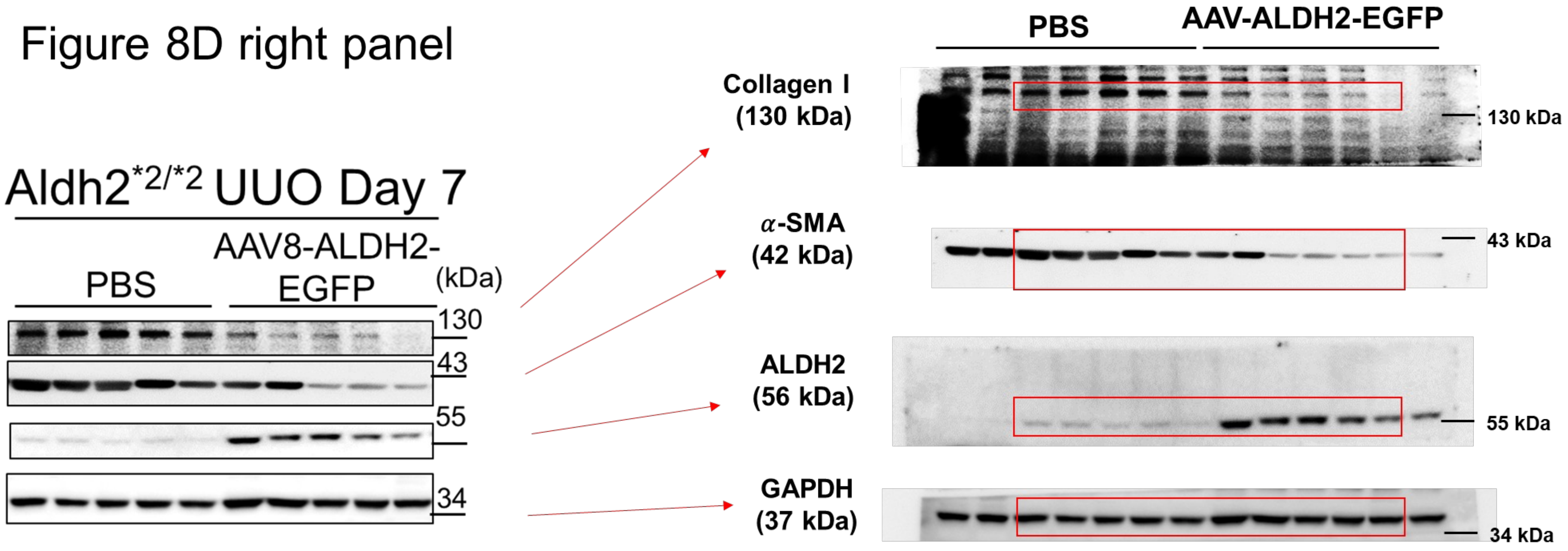

Supplement: Unedited blot and gel images [file jciinsight-9-179871-s021.pdf]
